# New Phenylpropanoid and Coumarin Glycosides from the Stems of *Hydrangea paniculata* Sieb

**DOI:** 10.3390/molecules22010133

**Published:** 2017-01-18

**Authors:** Jie Ma, Chuang-Jun Li, Jing-Zhi Yang, Hua Sun, Dong-Ming Zhang

**Affiliations:** State Key Laboratory of Bioactive Substance and Function of Natural Medicines, Institute of Materia Medica, Chinese Academy of Medical Sciences and Peking Union Medical College, Beijing 100050, China; majie@imm.ac.cn (J.M.); lichuangjun@imm.ac.cn (C.-J.L.); yjzh@imm.ac.cn (J.-Z.Y.); sunhua@imm.ac.cn (H.S.)

**Keywords:** *Hydrangea paniculata*, phenylpropanoid glycoside, coumarin glycoside, hepatoprotective activity

## Abstract

A new phenylpropanoid glycoside (**1**), and two new coumarin glycosides (**2**, **3**), together with two known compounds (**4**, **5**), have been isolated from the stems of *Hydrangea paniculata* Sieb. Their structures have been determined by spectroscopic and chemical methods. Furthermore, compound **1** (50 μM) exhibited significant hepatoprotective activity against *N*-acetyl-p-aminophenol (APAP)-induced HepG2 cell damage *in vitro* assays.

## 1. Introduction

**Figure 1:**
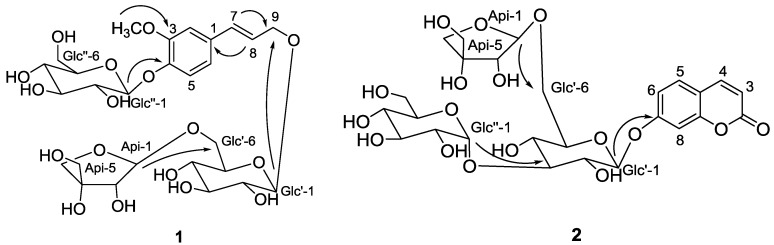

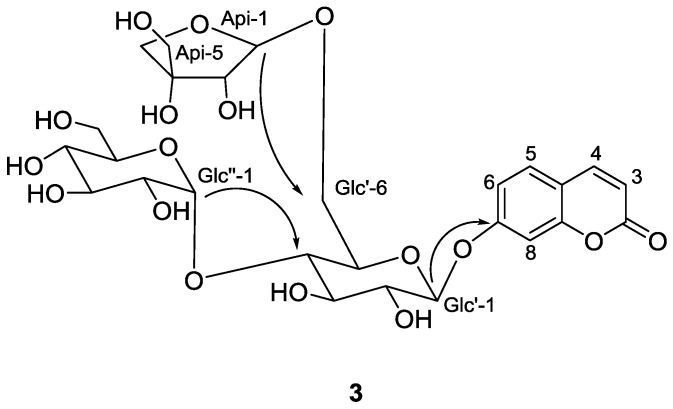
Structures and key HMBCs of compounds **1**–**3** from the stems of *Hydrangea paniculata* Sieb.

*Hydrangea paniculata* Sieb. (Saxifragaceae), widely distributed in southern part of China, has been employed to bring down fever, relieve sore throat, and treat malaria in folk medicine [1]. Our previous studies have found that the effective fraction of *H. paniculata* is used to make pharmaceutical compounds for the prevention and/or treatment of renal insufficiency, hypertension, and diabetic nephropathy [2,3]. In addition, further phytochemical investigations of this plant have led to the identification of coumarin glycosides, iridoid glucosides and secoiridoids, which showed diverse biological activities, such as neuroprotective effects, hepatoprotective and suppressive diabetic nephropathy [4,5,6]. As part of our ongoing research to identify bioactive constituents, a detailed phytochemical investigation on the water extract of the stems of *H. paniculata* was carried out. We reported herein the isolation and structural characterization of a new phenylpropanoid glycoside and two new coumarin glycosides (**1**–**3**), along with two known constituents, coniferinoside (**4**) and isoconferinoside (**5**) (Figure 1). The evaluations of their hepatoprotective activity were also displayed in this paper.

## 2. Results and Discussion

### 2.1. Purification and Characterization

Compound **1** was obtained as amorphous white power. The molecular formula, C_27_H_40_O_17_, was deduced from its HR-ESI-MS (*m*/*z* 659.2171, [M + Na]^+^, calc. 659.2158) with 8 degrees of unsaturation. The IR spectrum indicated the presence of an OH group (3382 cm^−1^) and an aromatic ring (1512 cm^−1^). The ^1^H-NMR spectrum (Table 1) showed three aromatic proton signals forming an ABX system at δ_H_ 7.07 (d, *J* = 1.5 Hz, H-2), 7.01 (d, *J* = 8.5 Hz, H-5) and 6.91 (dd, *J* = 8.5 Hz, 1.5, H-6) and two trans olefinic protons at δ_H_ 6.58 (d, *J* = 16.0 Hz, H-7) and 6.26 (dt, *J* = 16.0 Hz, 6.0, H-8), which suggested the present of one 1,3,4-trisubstituted phenylproanoid moiety. In addition, the ^1^H and ^13^C-NMR spectra exhibited resonances for two glucopyranosyl moieties δ_H_4.20 (d, *J* = 7.5 Hz, H-1′); δ_C_ 102.0 (C-1′) and 4.89 (d, *J* = 6.5 Hz, H-1′′); δ_C_ 100.0 (C-1′′) and a characteristic anomeric proton signal for apiofuranosyl moiety at δ_H_ 4.88 (d, *J* = 3.0, H-Api-1). The assignments of all proton and carbon signals were achieved with the help of ^1^H-^1^H-COSY, TOCSY, HSQC and HMBC experiments (see Supplementary Materials). Acid hydrolysis of **1** yielded d-glucose and d-apiose by GC analysis. The coupling constants (6–8 Hz) of the anomeric protons of the glucopyranosyl units as well as the chemical shift (δ_C_ 109.4) of the anomeric carbon of the apiofuranosyl unit demonstrated that all sugar units were β-anomeric configurations [7]. The linkages of the sugars and the sugars with the aglycone were established from the following HMBC correlations: δ_H_ 4.88 (H-api-1) correlated with δ_C_ 67.8 (C-6′), δ_H_ 4.20 (H-1′) to δ_C_ 68.8 (C-9) and 4.89 (H-1″) to δ_C_ 146.4 (C-4) (see Figure 1). Furthermore, the three-bond long-range HMBCs (Figure 1) between δ_H_ 3.77 (s, MeO-C-3) and δ_C_ 149.1 (C-3) confirmed that the MeO group was located at C-3. Thus, compound **1** was determined as conferyl alcohol-4-*O*-β-d-glucopyranoside-9-*O*-β-d-apio furanosyl (1 → 6)-β-d-glucopyranoside.

Compound **2** was isolated as amorphous white solid and had a molecular formula C_26_H_34_O_17_, as determined by HR-ESI-MS (*m*/*z* 641.1685 ([M + Na]^+^; calc. 641.1688). The UV spectrum showed absorbance at λ_max_ 204, 291 and 318 nm, and the IR spectrum exhibited the presence of a hydroxyl (3382 cm^−1^), carbonyl moiety (1708 cm^−1^) and aromatic ring (1617, 1072 cm^−1^). Three ABX-type coupled aromatic protons at δ_H_ 7.64 (d, *J* = 8.4 Hz, H-5), 7.01 (dd, *J* = 8.4 Hz, 1.8, H-6) and 7.03 (d, *J* = 1.8 Hz, H-8) and two coupled olefinic protons at δ_H_ 6.63 (d, *J* = 9.6 Hz, H-3) and 7.99 (d, *J* = 9.6 Hz, H-4), as well as three anomeric protons at δ_H_ 5.10 (d, *J* = 7.2 Hz, H-1′), 5.01 (d, *J* = 3.0 Hz, H-1″) and 4.80 (d, *J* = 3.0 Hz, H-Api-1) were observed in the ^1^H-NMR spectrum, suggesting the presence of 7-substituted coumarin aglycone [8] and three sugar moieties in **2**. Collectively, the NMR data indicated that the molecular structure of **2** was similar to umbelliferone 7-*O*-β-d-glucopyranosyl(1 → 3)-[β-d-apiofuranosyl(1 → 6)]-β-d-glucopyranoside [5], except for α-anomeric configuration of the terminal glucose (Glc′′). The α-orientation of the anomeric proton was assigned on the basis of the small *J* values (3.0 Hz) and an upfield shift of C-1 (+4.0 ppm) of Glc′′ (Table 1) [9]. The linkages of the sugars and the sugars with the aglycone were further confirmed by the following HMBC correlations: δ_H_ 4.80 (d, *J* = 3.0 Hz, H-Api-1)) to δ_C_ 67.2 (C-6′)), δ_H_ 5.01 (d, *J* = 3.0 Hz, H-1″) to δ_C_ 85.7 (C-3′), and δ_H_ 5.10 (d, *J* = 7.2 Hz, H-1′) to δ_C_ 160.1 (C-7) (Figure 1 and Table 1). After acid hydrolysis of 1, d-glucose and d-apiose was detected from the hydrolysate by GC analysis. Therefore, the structure of compound **2** was established as umbelliferone 7-*O*-α*-*d-glucopyranosyl(1 → 3)-[β-d-apiofuranosyl(1 → 6)]-β-d-glucopyranoside.

Compound **3** exhibited the same molecular formula, C_26_H_34_O_17_, as **2**, based on the HR-ESI-MS ion at *m*/*z* 641.1699 [M + Na]^+^ and the ^13^C-NMR data. The NMR spectra (Table 1) analysis of **3** indicated that it was also 7-substituted coumarin with two glucopyranosyl units and an apiofuranosyl unit. The major difference among them was the interglycosidic linkages. This assignment was confirmed by the HMBC correlations from δ_H_ 4.84 (d, *J* = 2.8 Hz, H-Api-1) to δ_C_ 67.6 (C-6′), δ_H_ 4.99 (d, *J* = 2.8 Hz, H-1″) to δ_C_ 80.5 (C-4′), and δ_H_ 5.08 (d, *J* = 7.8 Hz, H-1′) to δ_C_ 160.4 (C-7)) (Figure 1 and Table 1). The anometic proton of the terminal glucose was determined as α-configuration because of its ^3^*J*_H__-1,H__-2_ coupling constant (2.8 Hz). The other anomeric protons were β-orientation for the *J* values (2.8 and 7.8 Hz). Therefore, the structure of **3** was identified to be umbelliferone 7-*O*-α-d-glucopyranosyl(1 → 4)-[β-d-apiofuranosyl(1 → 6)]-β-d-glucopyranoside.

### 2.2. Structure Identification of the Known Isolates

The two known compounds were identified as coniferinoside (**4**) [10] and isoconferinoside (**5**) [11] based on the gCOSY, HSQC, and HMBC spectra, as well as on comparison with those previously reported physicochemical values in the literature.

### 2.3. Hepatoprotective Effect of Compounds ***1**–**3***

Compounds **1**–**3** were tested for their hepatoprotective activities against *N*-acetyl-p-amino phenol (APAP)-induced toxicity in HepG2 (human hepatocellular liver carcinoma cell line) cells, using the hepatoprotective activity drug bicyclol as the positive control [12]. At 50 μM, compound **1** reduced APAP-induced HepG2 cells damage by increasing the survival rate from 42.01% (*p* < 0.001) to 51.07% (*p* < 0.05), while the positive control bicyclol gave a 53.16% (*p* < 0.01) survival rate at 10 μM. However, survival rates of coumarin glycosides **2** and **3** were at 42.84% and 31.43%, respectively.

## 3. Experimental Section

### 3.1. General

The IR data were recorded on a Nicolet 5700 spectrometer （Thermo Nicolet Corporation, Madison, SD, USA） using an FT-IR microscope transmission method, and the UV spectra were scanned by a Jasco V650 spectrophotometer (Jasco Corporation, Tokyo, Japan). The NMR spectra were acquired on VNS-600 (Varian Medical Systems, Inc., Palo Alto, CA, USA), Bruker AV500-III and AVIII HD600 spectrometers (Bruker Corporation, Karlsruhe, Germany) with TMS as the internal standard. HR-ESI-MS were obtained on a Finnigan LTQ FT mass spectrometer (Thermo Fisher Scientific, Waltham, MA, USA) and ESI-MS on an Agilent 1100 series LC/MSD Trap-SL mass spectrometer (Agilent Technologies, Santa Clara, CA, USA). GC was performed using an Agilent Technologies 7890A instrument. Reversed-phase silica MPLC was conducted with pumps C-605 (Buchi, Flawil, Swizerland), a UV photometer C-635 (Buchi), a fractioncollector C-660 (Buchi), and an ODS column (6 cm × 45 cm, 50 μm, 800 g). Preparative HPLC was performed on a Shimadzu LC-6AD instrument (Shimadzu, Kyoto, Japan) with a SPD-20A detector, using a YMC-Pack ODS-A column (YMC, Kyoto, Japan) (250 mm × 20 mm, 5 μm). Silica gel (200–300 mesh, Qingdao Marine Chemical Inc., Qingdao, China), sephadex LH-20 (GE Healthcare Bio-Sciences AB, Uppsala, Sweden) and ODS (50 μm, YMC, Kyoto, Japan) were used for column chromatography (CC). TLC was performed using glass precoated silica gel GF_254_ plates. Spots were visualized under UV light or by spraying with 10% sulfuric acid in ethanol followed by heating.

### 3.2. Plant Material

The stems of *H. paniculata* were collected from the County of Jinxiu, Guangxi Zhuang Autonomous Region, China, in May 2009, and was identified by Mr. Guangri Long (Liuzhou Forestry Bureau of Guangxi). A voucher specimen (ID-4645) has been deposited with the Institute of Materia Medica, Chinese Academy of Medical Sciences, Beijing.

### 3.3. Extraction and Isolation

The air-dried stems of *H. paniculata* (20 kg) were crushed to a coarse solid and refluxed with water (2 × 90 L, each for 3 h). The combined H_2_O extract was passed through a macroporous resin (HPD100, 30 L) column eluted with H_2_O (90 L) and 17% ethanol (150 L) successively. The ethanolic portion was dried with a rotary vacuum evaporator and the residue was refluxed with 95% ethanol. After evaporation of the ethanol *in vacuo*, the aqueous part (400 g) was subjected to a macroporous resin column (HP2MGL, 12 L) eluted with water and 20% EtOH. The latter portion (200 g, A) was subjected to column chromatography on silica gel (200–300 mesh, 10 cm × 100 cm, 2.8 kg) using a stepwise gradient elution of CHCl_3_–MeOH–H_2_O (80:20:2–70:30:5, *v*/*v*/*v*) to afford thirteen fractions (A1–A13). The last portion A13 (27 g) was further chromatographed over a sephadex LH-20 column (5 cm × 130 cm, 600 g) using 50% EtOH as a mobile phase to give five subfractions (A13a–A13e). Fraction A13b (9.5 g) was separated by reversed-phase silica MPLC with 10%–40% aqueous MeOH (25 mL/min, 6 h) to afford forty parts (A13b1-40). Fraction A13b21 (500 mg) was performed repeatedly on an RP-18 column (ODS, 50 μm, YMC) with MeOH–H_2_O (14:86, *v*/*v*) as the mobile phase to give **4** (5 mg) and **5** (4 mg). Fraction A13b31 (170 mg) was followed by repeated separation on an RP-18 column (ODS, 50 μm, YMC) with MeOH–H_2_O (14:86, *v*/*v*) as the mobile phase yielded **2** (14 mg) and **3** (13 mg), using phenyl column (50 μm, ZORBAX) with MeOH–H_2_O (9:91, *v*/*v*) as the mobile phase to get **1** (14 mg).

*Conferyl alcohol-4-O-β-d-glucopyranoside-9-O-β-d-apiofuranosyl(1* → *6)-β-d-glucopyranoside* (**1**): white, amorphous solid. ${\left[\alpha \right]}_{D}^{20}$ −117.4 (*c* 0.13, MeOH). UV (MeOH) λ_max_ (log ε): 211 (4.56), 260 (4.35), 298 (3.90) nm; IR (microscope) ν_max_ 3382, 2921, 1512, 1266, 1076, 636 cm^−1^. ^1^H-NMR (DMSO-*d*_6_, 500 MHz) and ^13^C-NMR (DMSO-*d*_6_, 125 MHz), see Table 1. HRESIMS *m*/*z* 659.2171 [M + Na]^+^ (calcd. for C_27_H_40_NaO_17_, 659.2158).

*Umbelliferone 7-O-α-d-glucopyranosyl(1* → *3)-[β-d-apiofuranosyl(1* → *6)]-β-d-glucopyranoside* (**2**): white, amorphous solid. ${\left[\alpha \right]}_{D}^{20}$ −35.4 (*c* 0.17, MeOH). UV (MeOH) λ_max_ (log ε): 204 (4.57), 291 (4.12), 318 (4.26) nm; IR (microscope) ν_max_ 3382, 2925, 1708, 1617, 1072, 1026 cm^−1^. ^1^H-NMR (DMSO-*d*_6_, 600 MHz) and ^13^C-NMR (DMSO-*d*_6_, 150 MHz), see Table 1. HRESIMS *m*/*z* 641.1685 [M + Na]^+^ (calcd. for C_26_H_34_NaO_17_, 641.1688).

*Umbelliferone 7-O-α-d-glucopyranosyl(1* → *4)-[β-d-apiofuranosyl(1* → *6)]-β-d-glucopyranoside* (**3**): white, amorphous solid; ${\left[\alpha \right]}_{D}^{20}$ −50.6 (*c* 0.15, MeOH). UV (MeOH) λ_max_ (log ε): 204 (4.64), 291 (4.12), 318 (4.25) nm; IR (microscope) ν_max_ 3359, 2923, 1708, 1618, 1026 cm^−1^. ^1^H-NMR (DMSO-*d*_6_, 600 MHz) and ^13^C-NMR (DMSO-*d*_6_, 150 MHz), see Table 1. HRESIMS *m*/*z* 641.1699 [M + Na]^+^ (calcd. for C_26_H_34_NaO_17_, 641.1688).

### 3.4. Acid Hydrolysis and Sugar Analysis

Each compound (2 mg) was dissolved in 2 mL 2 M HCl and refluxed at 80 °C for 5 h. The reaction mixture was extracted with EtOAc (3 × 1 mL), and the aqueous layer was evaporated to give a mixture of monosaccharides. The residue was further dissolved in anhydrous pyridine (1 mL) followed by the addition of 2 mg l-cysteine methyl ester hydrochloride (J&K Scientific Ltd., Beijing, China; 99%). After heating at 60 °C for 2 h, the solvent was eliminated under N_2_, and 0.2 mL trimethylsilylimidazole (J&K Scientific Ltd., 99%) was added. Then, the mixture was heated at 60 °C for another 2 h and partitioned with *n*-hexane and water (2 mL each). The organic layer was analyzed by GC. The authentic samples were treated with the same method, d-glucose and d-apiose in a ratio of 2:1 were detected from **1** to **3**.

### 3.5. Hepatoprotective Activity Assay

Human HepG2 hepatoma cells were cultured in DMEM medium supplemented with 10% fetal calf serum, 100 U/mL penicillin, and 100 μg/mL streptomycin at 37 °C in a humidified atmosphere of 5% CO_2_ + 95% air. The cells were then passaged by treatment with 0.25% trypsin in 0.02% EDTA. The MTT assay was used to assess the cytotoxicity of test samples. The cells were seeded in 96-well multiplates. After an overnight incubation at 37 °C with 5% CO_2_, 50 μM test samples and APAP (final concentration of 8 mM) were added into the wells and incubated for another 48 h. Then, 100 μL of 0.5 mg/mL MTT was added to each well after the withdrawal of the culture medium and incubated for an additional 4 h. The resulting formazan was dissolved in 150 μL of DMSO after aspiration of the culture medium. The plates were placed on a plate shaker for 30 min and read immediately at 570 nm using a microplate reader (μquant Bio-tek, Bio-tek instruments, inc., Winooski, VT, USA) to dertermine the OD value [12].

## 4. Conclusions

In summary, this work described the isolation and structure identification of one new phenylpropanoid glycoside (**1**) and two new (**2**, **3**) coumarin glycosides along with two known compounds (**4**, **5**) from the stems of *Hydrangea paniculata* Sieb., and their hepatoprotective activities against APAP-induced toxicity in HepG2 cells. Compound **1**, phenylpropanoid glycoside including d-apiofuranosyl moiety, was found in this plant for the first time. In addition, it was unusual that sugar chains of compounds **2** and **3** contained α-d-glucose in the plant.

## Figures and Tables

**Table 1:** ^1^H- and ^13^C-NMR spectroscopic data for compounds **1**–**3**. δ in ppm, *J* in Hz.

Position	1 ^a^		2 ^b^		3 ^b^	
	δ_H_	δ_C_	δ_H_	δ_C_	δ_H_	δ_C_
1		130.6 (s)				
2	7.07 (d, 1.5)	109.9 (d)		160.3 (s)		160.6 (s)
3		149.1 (s)	6.63 (d, 9.6)	113.4 (d)	6.32 (d, 9.6)	113.7 (d)
4		146.4 (s)	7.99 (d, 9.6)	144.3 (d)	7.98 (d, 9.6)	144.6 (d)
5	7.01 (d, 8.5)	115.2 (d)	7.64 (d, 8.4)	129.6 (d)	7.65 (d, 9.0)	129.9 (d)
6	6.91 (dd, 8.5, 1.5)	119.5 (d)	7.01 (dd, 8.4, 1.8)	113.5 (d)	7.02 (dd, 9.0, 2.4)	113.8 (d)
7	6.58 (d, 16.0)	131.7 (d)		160.1 (s)		160.4 (s)
8	6.26 (dt, 16.0, 6.0)	124.4 (d)	7.03 (d, 1.8)	103.4 (d)	7.03 (d, 2.4)	103.7 (d)
9	4.38 (dd, 13.5, 6.0); 4.16 (dd, 13.5, 6.0)	68.8 (t)		155.1 (s)		155.4 (s)
10	3.77(s, MeO-C(3))	55.7 (q)		113.4 (s)		113.6 (s)
Glc′						
1	4.20 (d, 7.5)	102.0 (d)	5.10 (d, 7.2)	99.8 (d)	5.08 (d, 7.8)	99.9 (d)
2	2.98 ^c^	73.5 (d)	3.42 ^c^	71.7 (d)	3.48 ^c^	73.9 (d) ^c^
3	3.14 ^c^	76.7 (d)	3.48 (m)	85.7 (d)	3.56 ^c^	76.3 (d)
4	2.98 ^c^	70.4 (d)	3.41 ^c^	69.4 (d)	3.38 ^c^	80.5 (d)
5	3.25 ^c^	75.7 (d)	3.69 (m)	74.9 (d)	3.78 (m)	74.2 (d)
6	3.86 (dd, 11.5, 4.5); 3.41 ^c^	67.8 (t)	3.47 (m);3.85 (dd,11.5,4.2)	67.2 (t)	3.88 ^c^; 3.59 (m)	67.6 (t)
Glc′′						
1	4.89 (d, 6.5)	100.0 (d)	5.01(d, 3.0)	100.3 (d)	4.99 (d, 2.8)	101.7 (d)
2	3.24 ^c^	73.3 (d)	3.24 ^c^	72.9 (d)	3.24 (m)	72.9 (d) ^c^
3	3.25 ^c^	77.0 (d)	3.45 ^c^	73.5 (d)	3.36 ^c^	73.7 (d)
4	3.14 ^c^	69.7 (d)	3.11 (m)	70.1 (d)	3.12 (m)	70.0 (d)
5	3.25 ^c^	77.1 (d)	3.73 (m)	72.6 (d)	3.47 (m)	72.9 (d)
6	3.65 (dd, 12.0, 5.0); 3.42 ^c^	60.7 (t)	3.61 (dd,12.0, 4.8); 3.45 ^c^	60.7 (t)	3.64 ^c^; 3.50 ^c^	60.9 (t)
Api						
1	4.88 (d, 3.0)	109.4 (d)	4.80 (d, 3.0)	109.4 (d)	4.84 (d, 2.8)	109.7 (d)
2	3.76 (d, 3.0)	76.0 (d)	3.75 (d, 3.0)	76.0 (d)	3.73 (d, 2.8)	76.4 (d)
3		78.9 (s)		78.8 (s)		79.1 (s)
4	3.86 (d, 9.5); 3.58 (d, 9.5)	73.3 (t)	3.88 (d, 9.6); 3.58 (d, 9.6)	73.5 (t)	3.87 (d, 9.6) ^c^; 3.57 (d, 9.6) ^c^	73.8 (t)
5	3.32 (m)	63.1 (t)	3.34 (m)	63.2 (t)	3.34 (m)	63.6 (t)

^a^ ^1^H-NMR data (δ_H_) were measured at 500 MHz, ^13^C-NMR data (δ_C_) were measured at 125 MHz; ^b^ ^1^H-NMR data (δ_H_) were measured at 600 MHz, ^13^C-NMR data (δ_C_) were measured at 150 MHz; ^c^ overlapping signals.

## Supplementary Materials

The following are available online at http://www.mdpi.com/1420-3049/22/1/133/s1, Figures S1–S30.
