# Supplementary material for: New Phenylpropanoid and Coumarin Glycosides from the Stems of Hydrangea paniculata Sieb
Source: Molecules. 2017 Jan 18;22(1):133. doi: 10.3390/molecules22010133 (PMC6155810; doi:10.3390/molecules22010133)
Supplement: Supplementary file 1 [file molecules-22-00133-s001.pdf]

Molecules **2017**, *22*, 133; doi:10.3390/molecules22010133

Supplementary Materials: New Phenylpropanoid and Coumarin Glycosides from the Stems of *Hydrangea paniculata* Sieb

Jie Ma, Chuang-Jun Li, Jing-Zhi Yang, Hua Sun and Dong-Ming Zhang

Qualitative Analysis Report

**Data Filename**	2015112602.d	**Sample Name**	HP-aa-9
**Sample Type**	Sample	**Position**	P1-C2
**Instrument Name**	Instrument 1	**User Name**	
**Acq Method**		**IRM Calibration Status**	Success
**DA Method**	TEST LCMS.m	**Comment**	

User Chromatograms

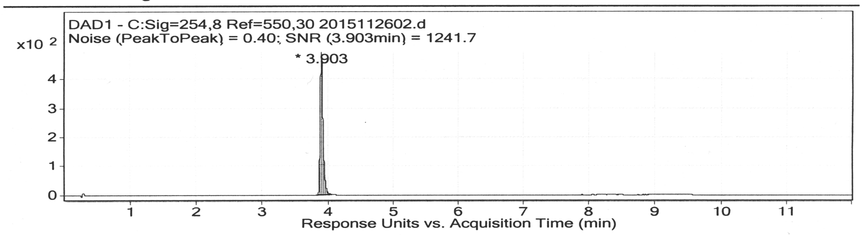

Integration Peak List

Peak	Start	RT	End	Height	Area	Area %	Signal To Noise
1	3.818	3.903	4.067	492.61	1552.58	100	1241.7

Fragmentor Voltage 120 **Collision Energy** 0 **Ionization Mode** ESI

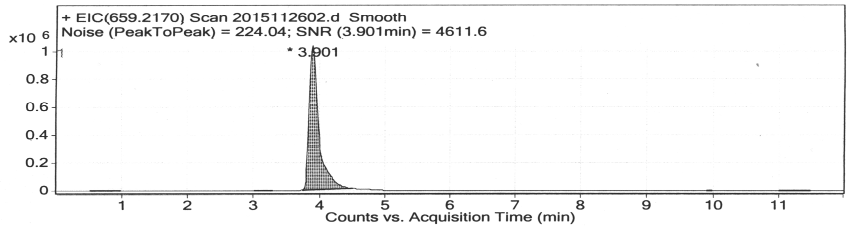

Integration Peak List

Peak	Start	RT	End	Height	Area	Area %	Signal To Noise
1	3.74	3.901	4.513	1033200	10626978	100	4611.6

User Spectra

Fragmentor Voltage	Collision Energy	Ionization Mode
120	0	Esi

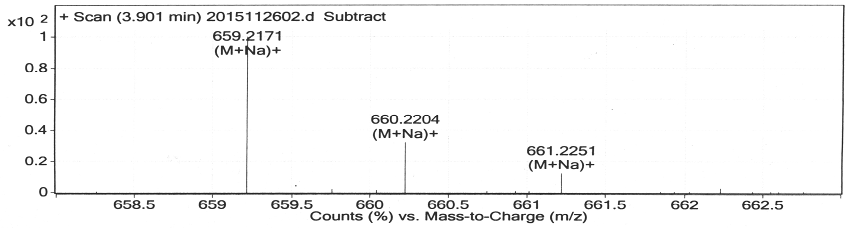

MS Formula Results: + Scan (3.901 min) Sub (2015112602.d)

m/z	Ion	Formula	Abundance
659.2171	(M+Na)+	C27 H40 Na O17	1314988

Best	Formula (M)	Ion Formula	Score	Cross Sco	Mass	Calc Mass	Calc m/z	Diff (ppm)	Abs Diff (ppm)	Mass Match	Abund Match	Spacing Match	DBE
☑	C27 H40 O17	C27 H40 Na O17	99.28		636.2279	636.2265	659.2158	-2.05	2.05	99.86	97.86	99.84	8
☐	C28 H36 N4 O13	C28 H36 N4 Na O13	99.19		636.2279	636.2279	659.2171	0.04	0.04	100	97.33	99.81	13
☐	C25 H40 N4 O13 S	C25 H40 N4 Na O13 S	99.17		636.2279	636.2313	659.2205	5.32	5.32	99.07	99.66	98.79	8
☐	C24 H44 O17 S	C24 H44 Na O17 S	99.17		636.2279	636.2299	659.2191	3.23	3.23	99.65	98.61	98.85	3
☐	C29 H40 N4 O8 S2	C29 H40 N4 Na O8 S2	99.14		636.2279	636.2288	659.218	1.38	1.38	99.94	98.57	98.24	12
☐	C28 H44 O12 S2	C28 H44 Na O12 S2	99.11		636.2279	636.2274	659.2166	-0.71	0.71	99.98	98.32	98.29	7
☐	C31 H40 O12 S	C31 H40 Na O12 S	99.08		636.2279	636.224	659.2133	-5.99	5.99	98.82	99.52	99.08	12
☐	C32 H36 N4 O8 S	C32 H36 N4 Na O8 S	99.08		636.2279	636.2254	659.2146	-3.91	3.91	99.5	98.42	99.03	17
☐	C37 H36 N2 O6 S	C37 H36 N2 Na O6 S	98.36		636.2279	636.2294	659.2186	2.43	2.43	99.81	95.23	99.23	21
☐	C41 H36 N2 O S2	C41 H36 N2 Na O S2	97.07		636.2279	636.2269	659.2161	-1.51	1.51	99.92	90.89	98.77	25
☐	C40 H32 N2 O6	C40 H32 N2 Na O6	96.88		636.2279	636.226	659.2153	-2.86	2.86	99.73	89.62	99.91	26
☐	C45 H32 O4	C45 H32 Na O4	95.47		636.2279	636.2301	659.2193	3.47	3.47	99.6	84.86	99.94	30
☐	C46 H28 N4	C46 H28 N4 Na	94.21		636.2279	636.2314	659.2206	5.56	5.56	98.98	81.49	99.92	35

Figure S1. HRESIMS spectrum of compound **1**.

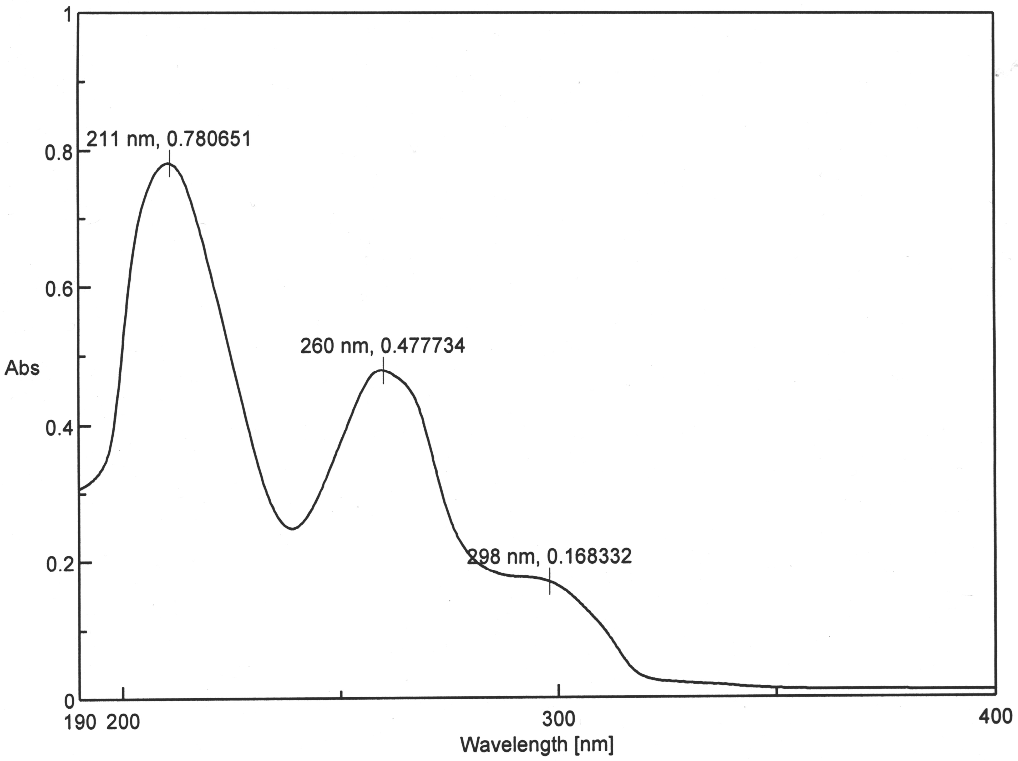

hp-aa-9

[Comment]

Sample Name	hp-aa-9
Comment	0.0135
User	
Division	UV
Company	324

[Measurement Information]

Instrument Name	V-650
Model Name	V-650
Serial No.	A034461150
Accessory	PSC-718
Accessory S/N	A001761114
Position	1
Cell Length	10 mm
Temperature	19.99 C
Control Sonsor	Holder
Monitor Sensor	Holder
Start Mode	Start immediately
Photometric Mode	Abs
Measurement range	400 - 190 nm
Data pitch	0.2 nm
Band width(UV/Vis)	2.0 nm
Response	Medium
Scanning speed	200 nm/min
Source Change	340 nm
Light Source	D2/WI
Filter Exchange	Step
Correction	Baseline

[Data Information]

Creation Date	2015-11-17 13:14
Data array type	Linear data array
Horizontal	Wavelength [nm]
Vertical	Abs
Start	400 nm
End	190 nm
Data pitch	0.2 nm
Data points	1051

Figure S2. UV spectrum of compound **1**.

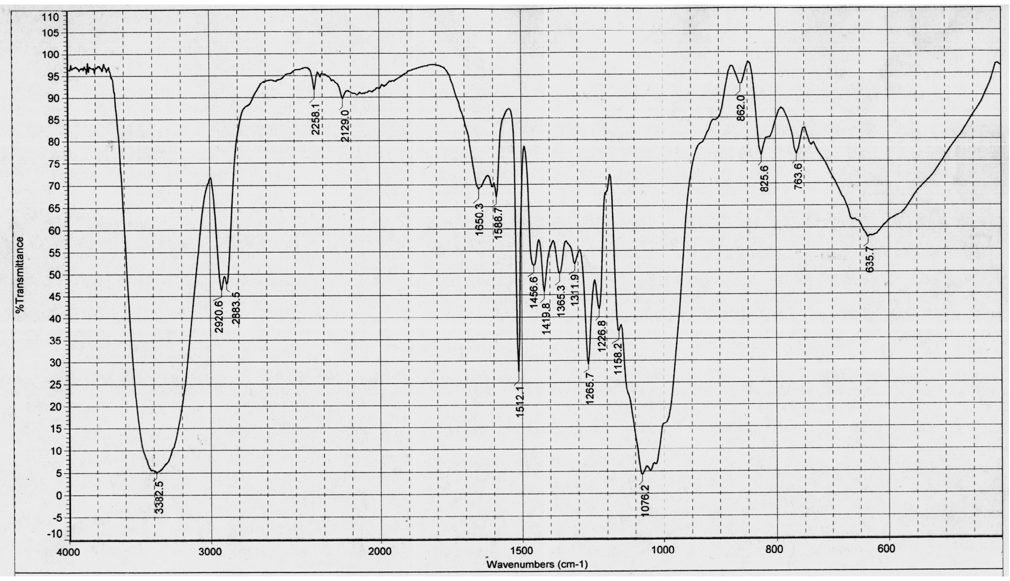

Figure S3. IR spectrum of compound **1**.

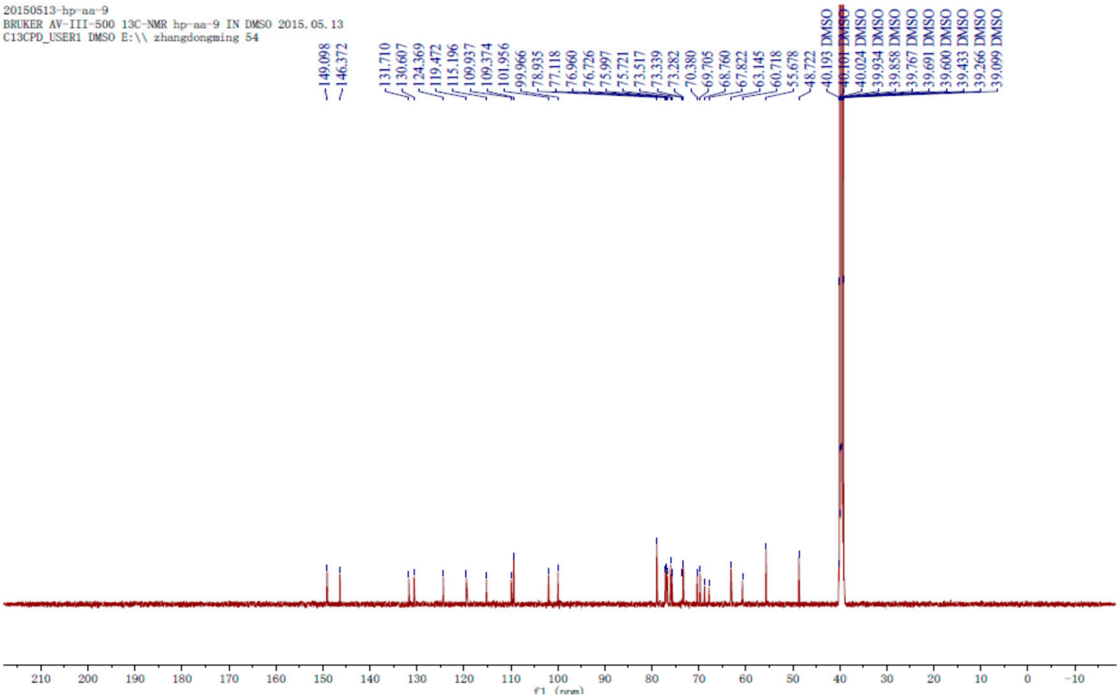

Figure S4. ^{13}C spectrum of compound **1**.

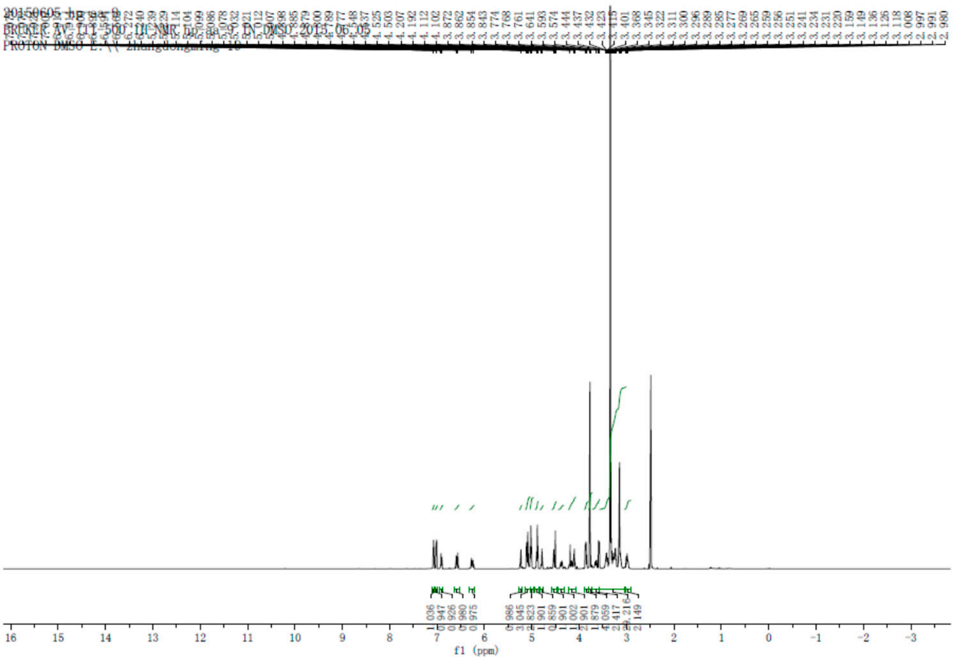

Figure S5. ^{1}H spectrum of compound **1**.

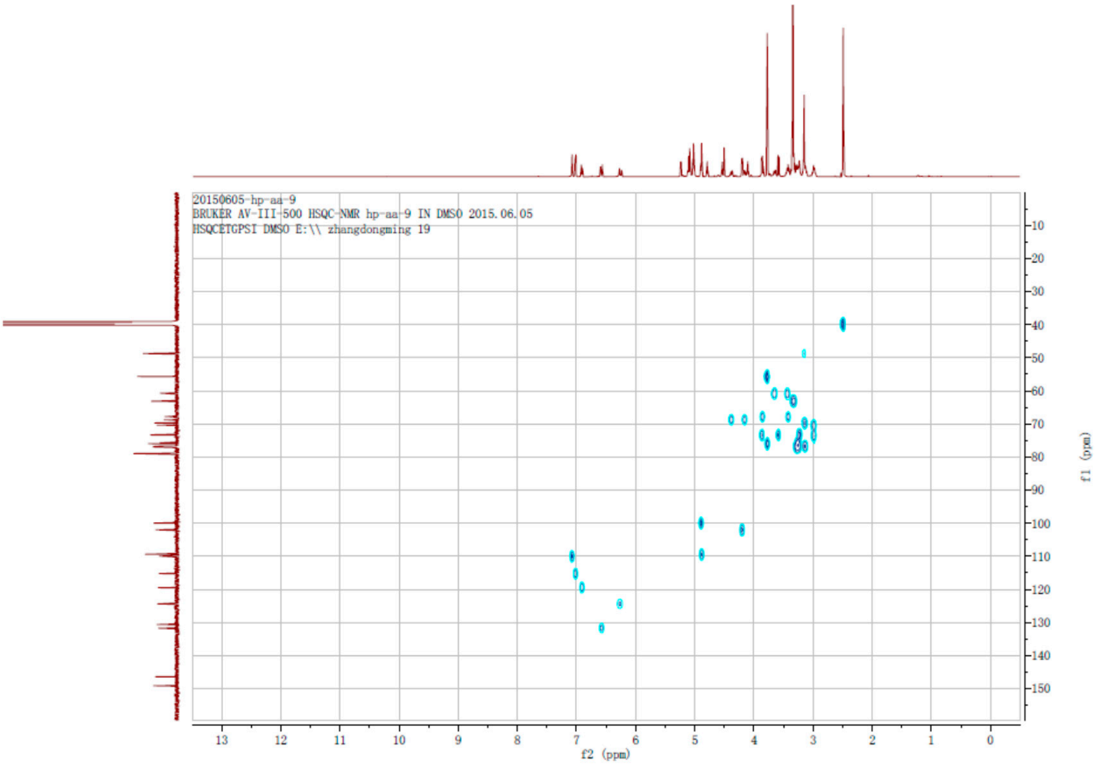

Figure S6. HSQC spectrum of compound **1**.

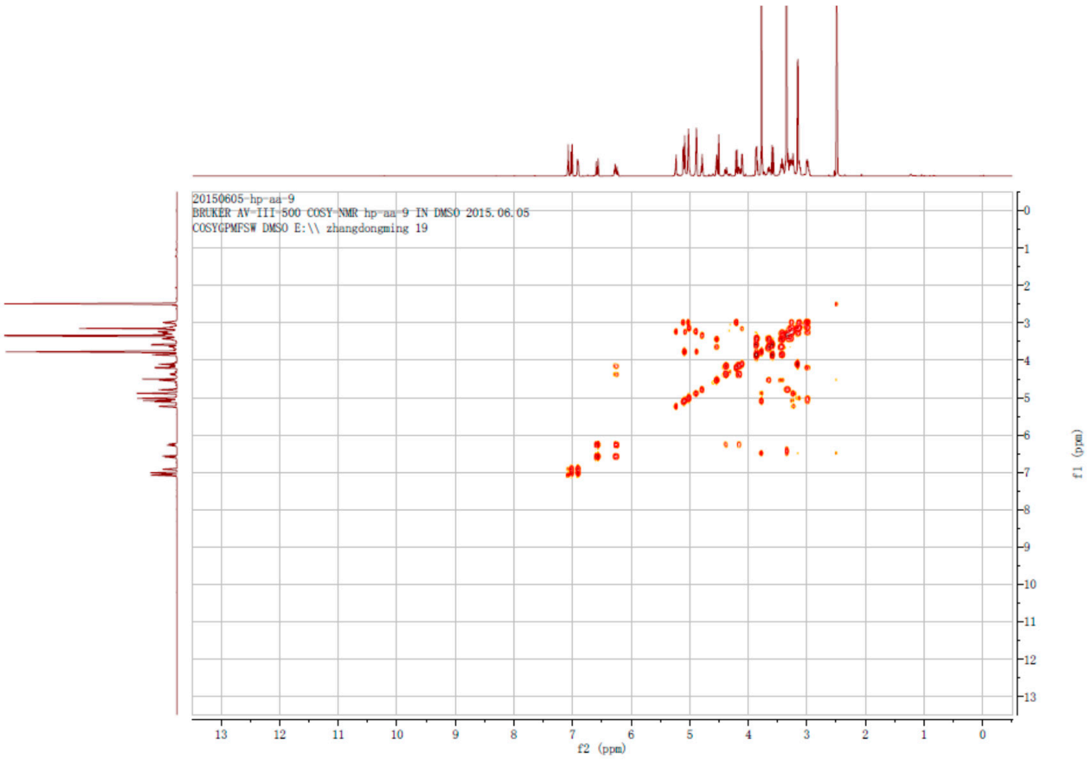

Figure S7. ^{1}H-^{1}H COSY spectrum of compound **1**.

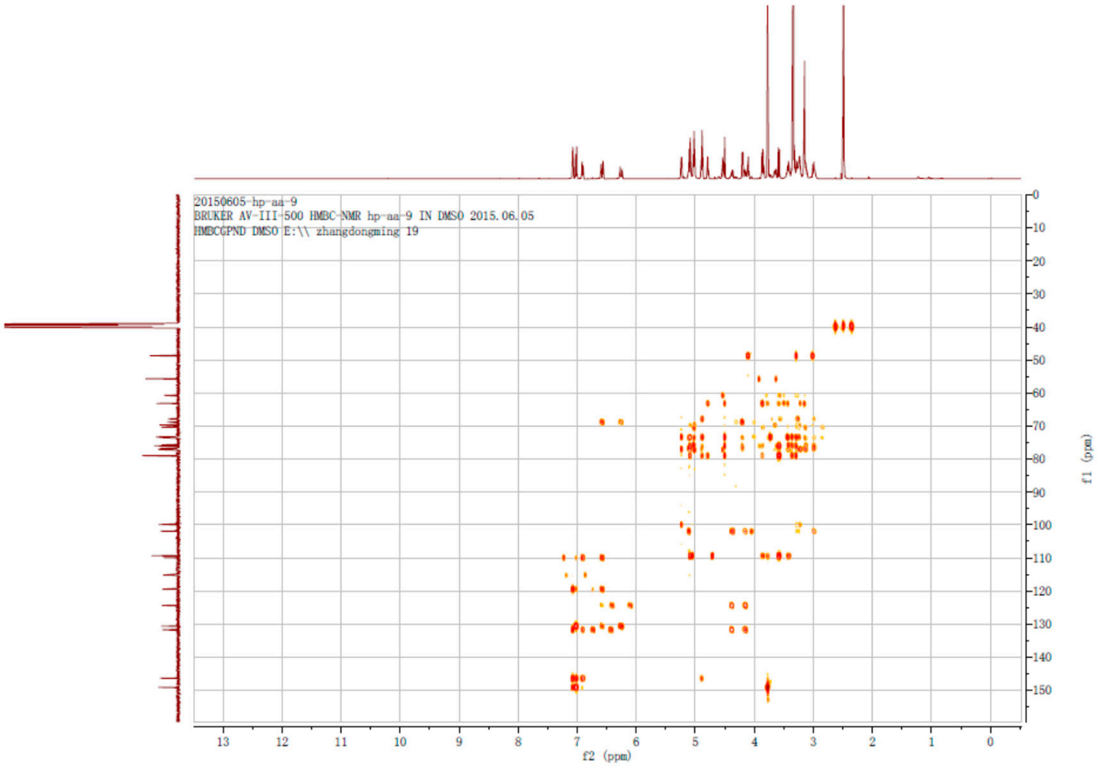

Figure S8. HMBC spectrum of compound **1**.

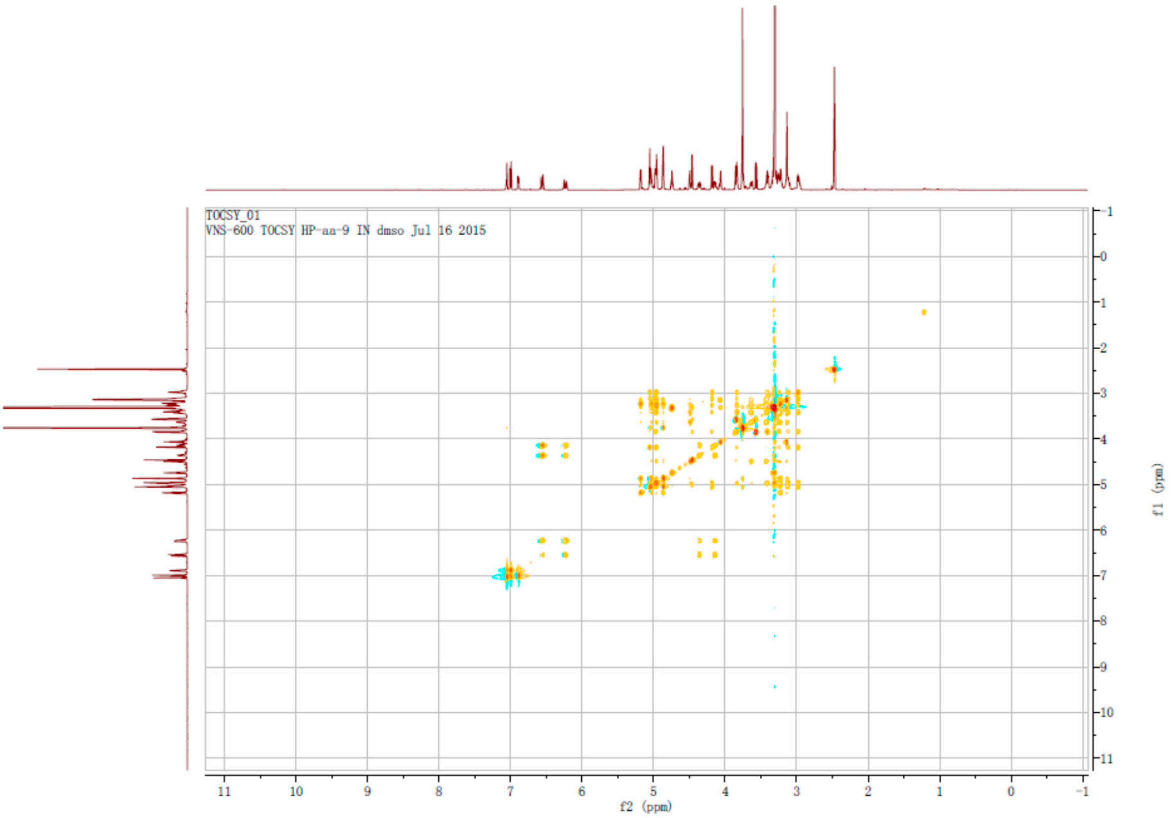

Figure S9. TOCSY spectrum of compound **1**.

Qualitative Analysis Report

**Data Filename**	2015112605.d	**Sample Name**	HP-aa-20
**Sample Type**	Sample	**Position**	P1-C5
**Instrument Name**	Instrument 1	**User Name**	
**Acq Method**		**IRM Calibration Status**	Success
**DA Method**	TEST LCMS.m	**Comment**	

User Chromatograms

Fragmentor Voltage 120 **Collision Energy** 0 **Ionization Mode** ESI

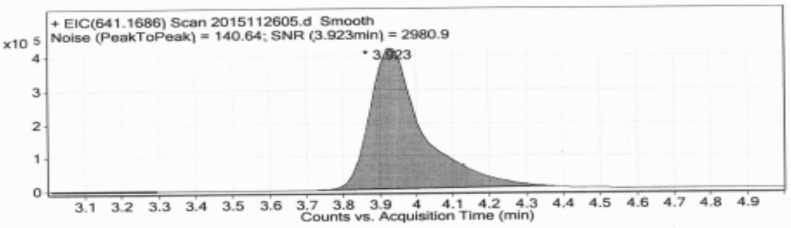

Integration Peak List

Peak	Start	RT	End	Height	Area	Area %	Signal To Noise
1	3.73	3.923	4.374	419232	4201443	100	2980.9

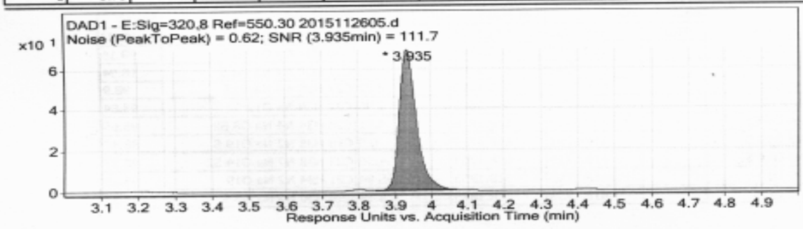

Integration Peak List

Peak	Start	RT	End	Height	Area	Area %	Signal To Noise
1	3.846	3.935	4.071	68.71	225.14	100	111.7

User Spectra

Fragmentor Voltage 120 **Collision Energy** 0 **Ionization Mode** Esi

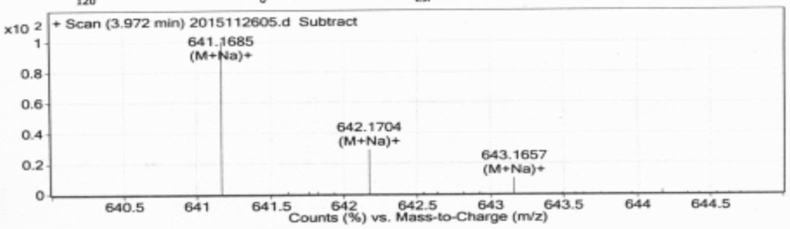

MS Formula Results: + Scan (3.972 min) Sub (2015112605.d)

m/z	Ion	Formula	Abundance
641.1685	(M+Na)+	C26 H34 Na O17	353113.1

Best	Formula (M)	Ion/Formula	Score	Cross Sco	Mass	Calc Mass	Calc m/z	Diff (ppm)	Abs Diff (ppm)	Mass Match	Abund Match	Spacing Match	DBE
☑	C26 H34 O17	C26 H34 Na O17	99.22		618.1792	618.1796	641.1688	0.57	0.57	99.99	99.25	97.63	10
☐	C30 H34 O12 S	C30 H34 Na O12 S	99.16		618.1793	618.1771	641.1663	-3.49	3.49	99.6	98.55	99.03	14
☐	C31 H30 N4 O8 S	C31 H30 N4 Na O8 S	98.94		618.1793	618.1784	641.1677	-1.33	1.33	99.94	97.14	99.11	19
☐	C27 H30 N4 O13	C27 H30 N4 Na O13	98.9		618.1793	618.1809	641.1702	2.72	2.72	99.76	98.41	97.76	15
☐	C27 H38 O12 S2	C27 H38 Na O12 S2	98.84		618.1793	618.1805	641.1697	1.96	1.96	99.87	96.52	99.57	9
☐	C28 H34 N4 O8 S2	C28 H34 N4 Na O8 S2	98.65		618.1793	618.1818	641.171	4.11	4.11	99.44	96.51	99.63	14
☐	C18 H38 N2 O19 S	C18 H38 N2 Na O19 S	98.47		618.1793	618.1789	641.1682	-0.5	0.5	99.99	95.18	99.39	1
☐	C22 H38 N2 O14 S2	C22 H38 N2 Na O14 S2	98.37		618.1793	618.1764	641.1657	-4.56	4.56	99.32	95.7	99.69	5
☐	C21 H34 N2 O19	C21 H34 N2 Na O19	98.29		618.1793	618.1756	641.1648	-5.94	5.94	98.84	97.71	97.89	6
☐	C36 H30 N2 O6 S	C36 H30 N2 Na O6 S	97.31		618.1793	618.1825	641.1717	5.18	5.18	99.12	92.97	98.89	23
☐	C35 H30 N4 O3 S2	C35 H30 N4 Na O3 S2	97.04		618.1793	618.1759	641.1652	-5.38	5.38	99.05	91.64	99.48	23
☐	C40 H30 N2 O S2	C40 H30 N2 Na O S2	96.05		618.1793	618.[illegible]8	641.1692	1.13	1.13	99.96	86.83	99.31	27
☐	C39 H26 N2 O6	C39 H26 N2 Na O6	95.93		618.1792	618.1791	641.1683	-0.26	0.26	100	88	97.33	28
☐	C43 H26 N2 O S	C43 H26 N2 Na O S	94.89		618.1793	618.1766	641.1658	-4.32	4.32	99.39	84.26	98.64	32

Figure S10. HRESIMS spectrum of compound **2**.

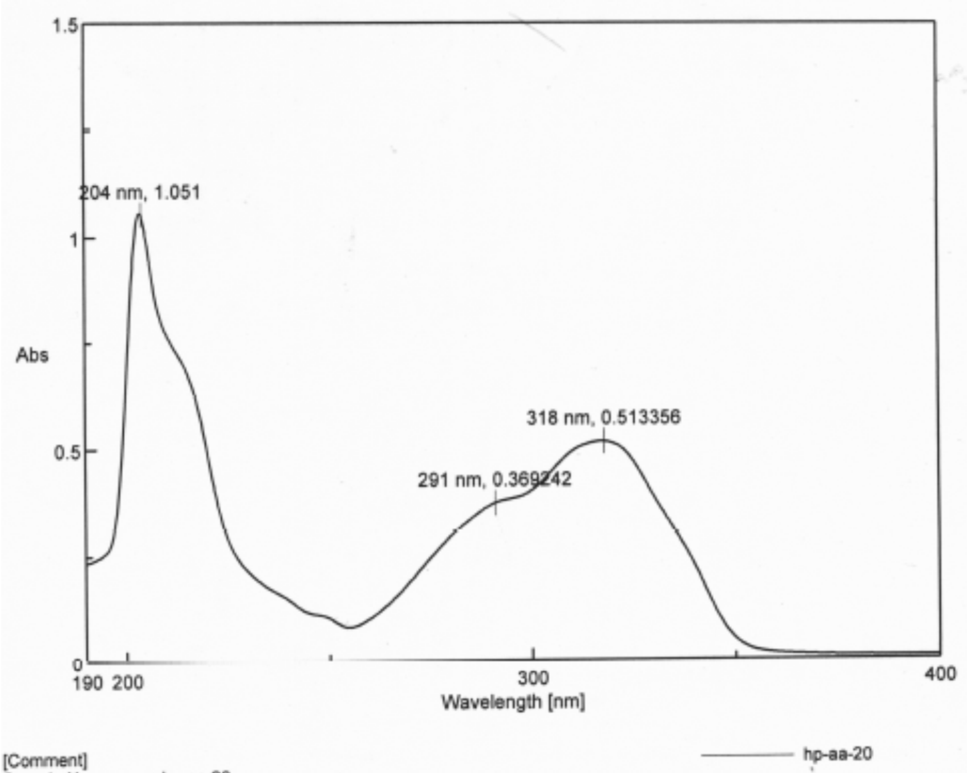

[Comment]
Sample Name hp-aa-20
Comment 0.02
User
Division UV
Company 324
[Measurement Information]
Instrument Name V-650
Model Name V-650
Serial No. A034461150

Accessory PSC-718
Accessory S/N A001761114
Position 1
Cell Length 10 mm
Temperature 19.98 C
Control Sonsor Holder
Monitor Sensor Holder
Start Mode Start immediately

Photometric Mode Abs
Measurement range 400 - 190 nm
Data pitch 0.2 nm
Band width(UV/Vis) 2.0 nm
Response Medium
Scanning speed 200 nm/min
Source Change 340 nm
Light Source D2/WI
Filter Exchange Step
Correction Baseline

[Data Information]
Creation Date 2015-11-17 12:10

Data array type Linear data array
Horizontal Wavelength [nm]
Vertical Abs
Start 400 nm
End 190 nm
Data pitch 0.2 nm
Data points 1051

Figure S11. UV spectrum of compound **2**.

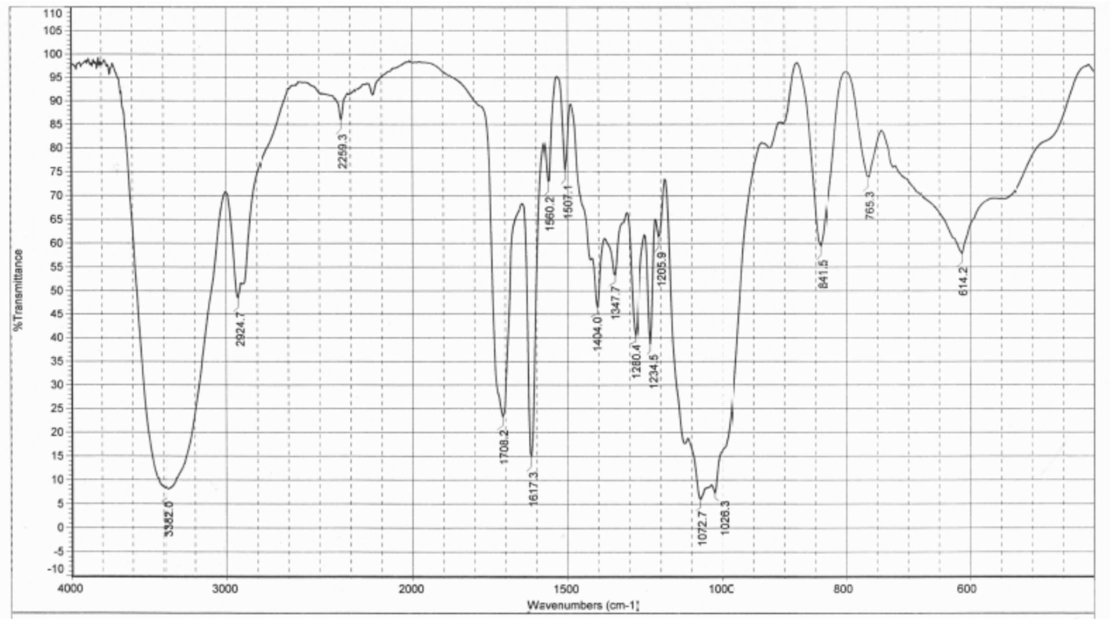

Figure S12. IR spectrum of compound **2**.

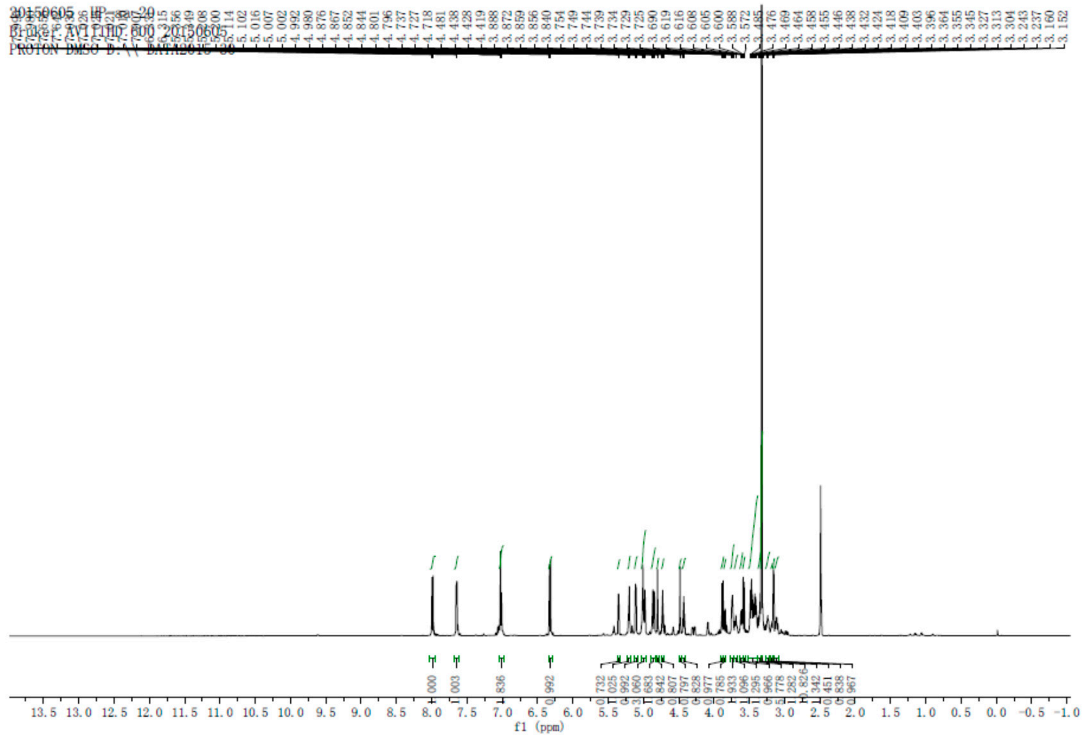

Figure S13. ^{1}H spectrum of compound **2**.

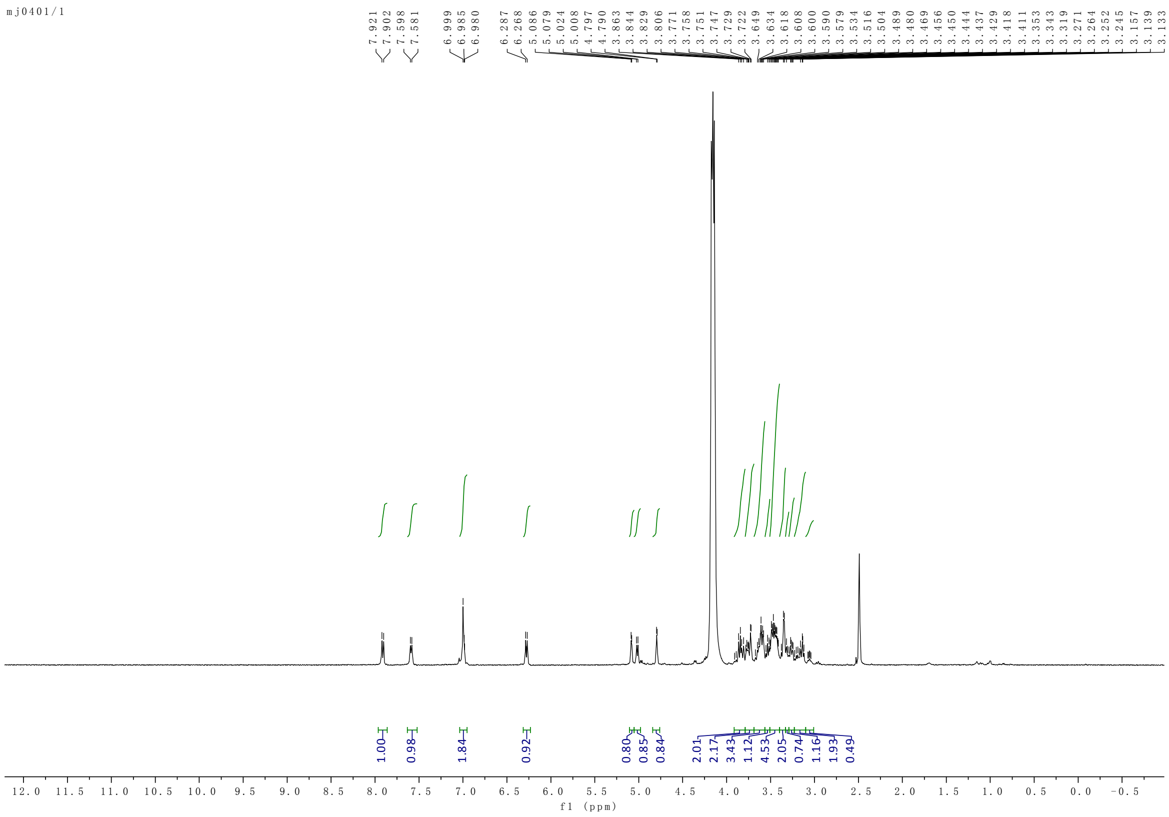

Figure S14. ^{1}H spectrum (in DMSO-d_6 + D_2O) of compound **2**.

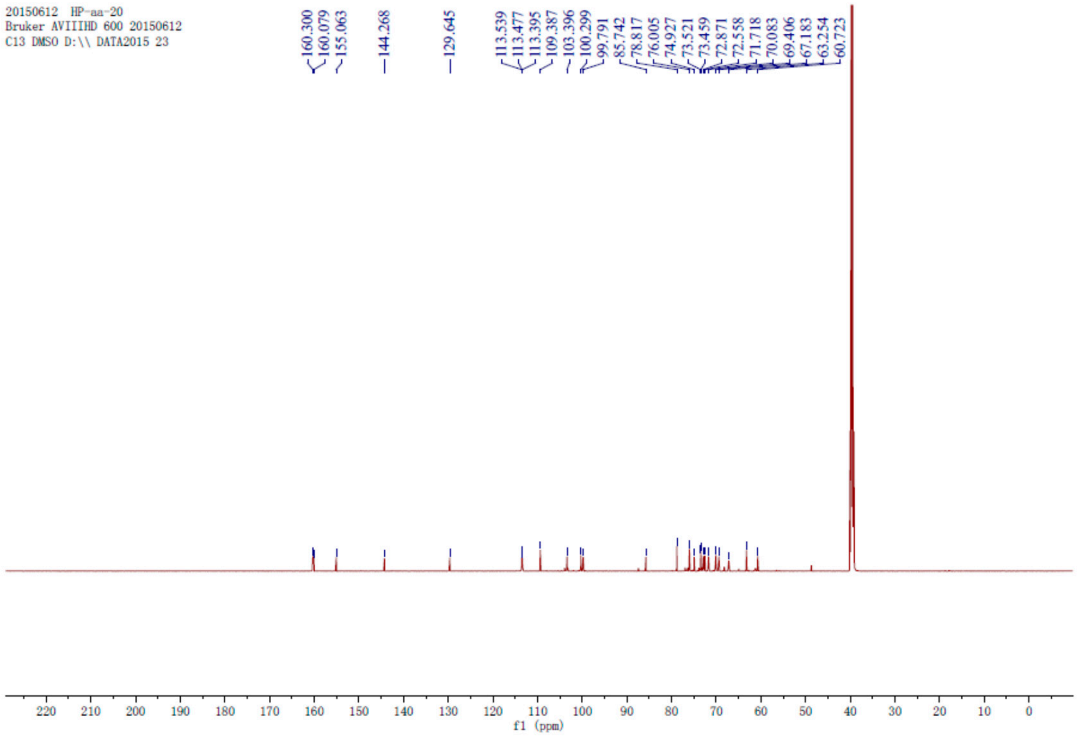

Figure S15. ^{13}C spectrum of compound **2**.

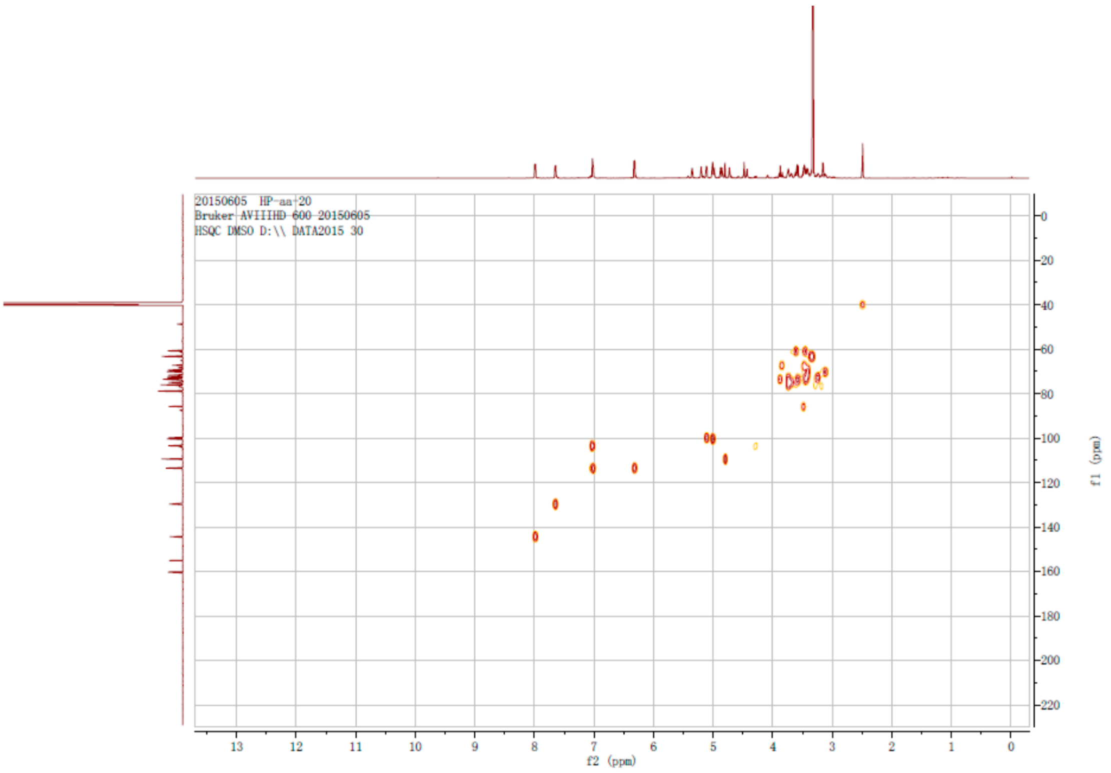

Figure S16. HSQC spectrum (in DMSO-d_6) of compound **2**.

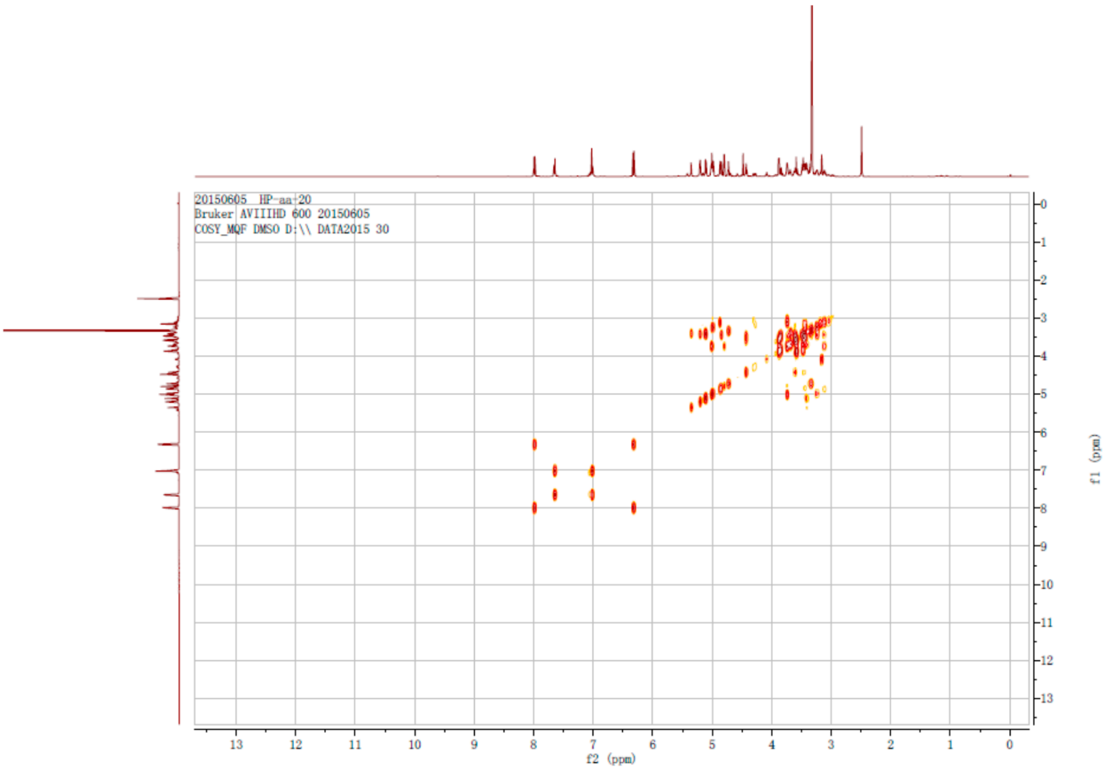

Figure S17. gCOSY spectrum of compound **2**.

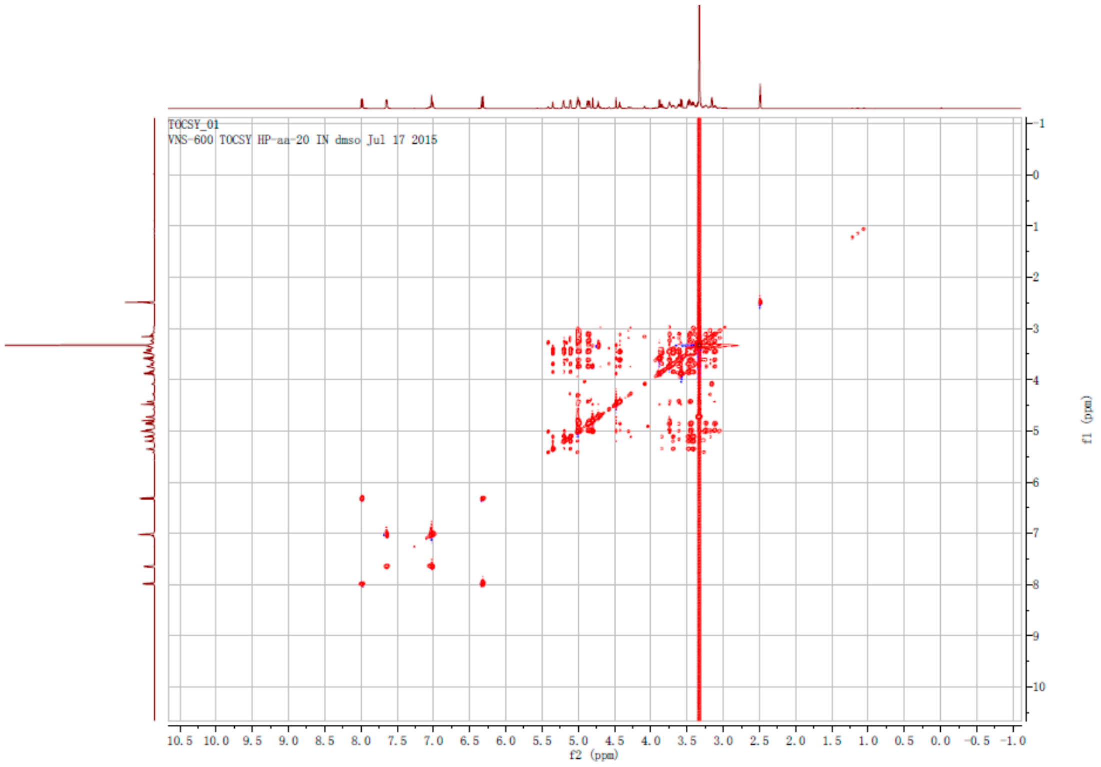

Figure S18. TOCSY spectrum of compound **2**.

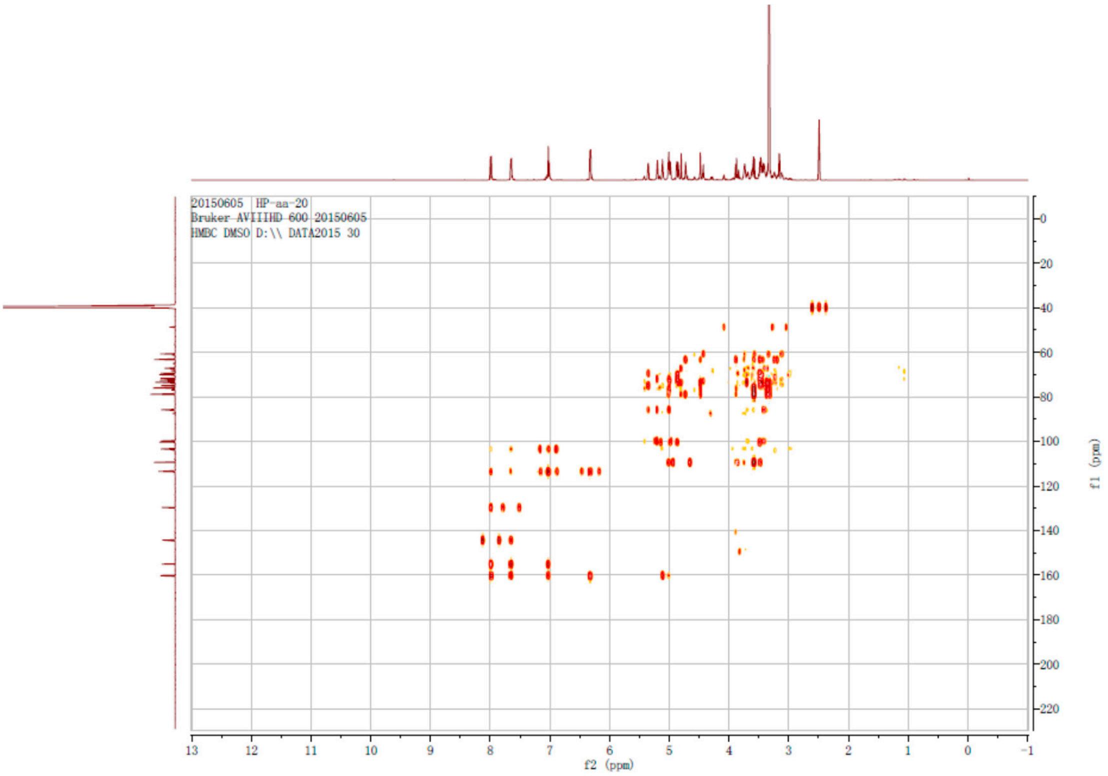

Figure S19. HMBC spectrum of compound **2**.

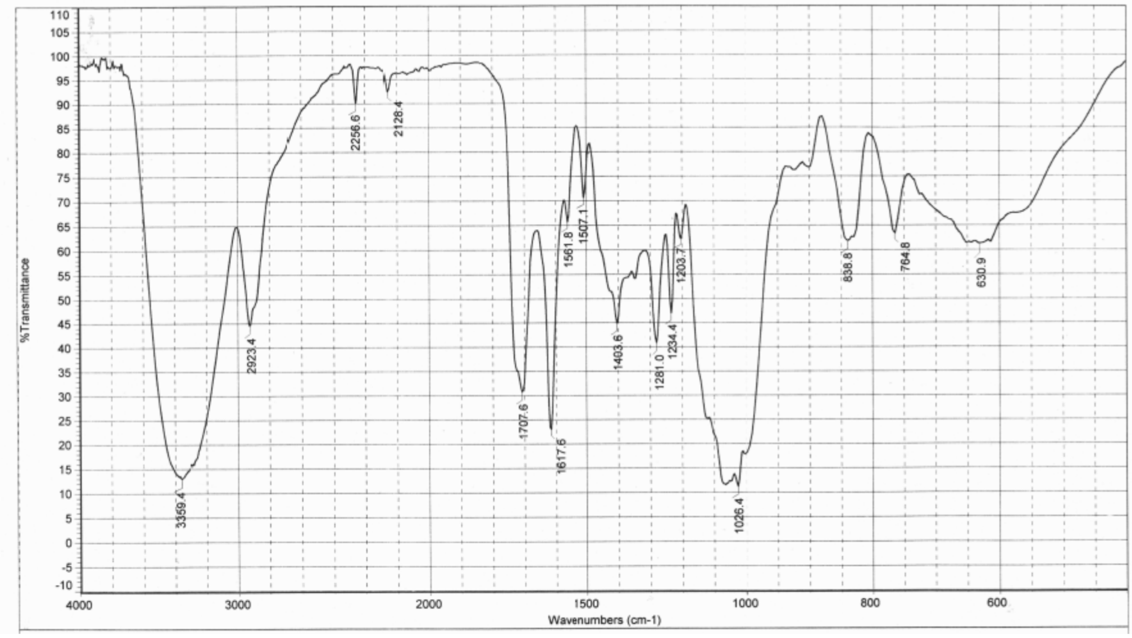

Figure S20. IR spectrum of compound **3**.

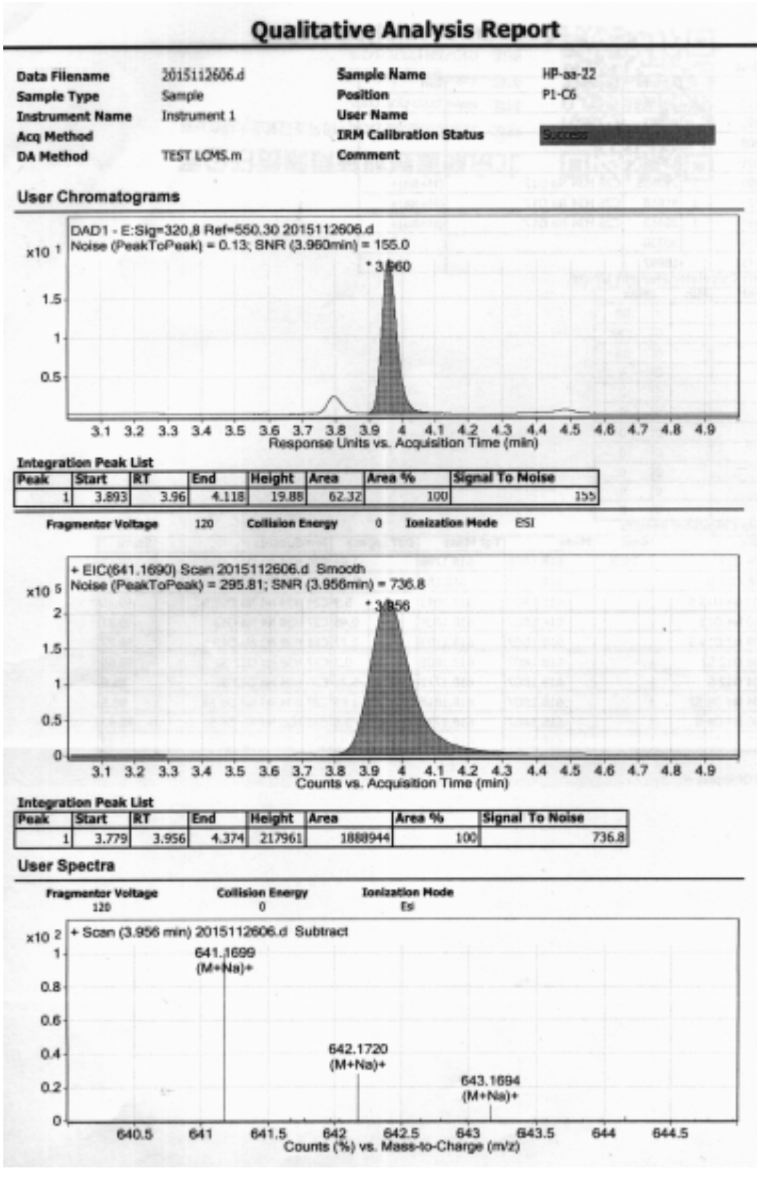

Qualitative Analysis Report

**Data Filename**	2015112606.d	**Sample Name**	HP-aa-22
**Sample Type**	Sample	**Position**	P1-C6
**Instrument Name**	Instrument 1	**User Name**	
**Acq Method**		**IRM Calibration Status**	Success
**DA Method**	TEST LCMS.m	**Comment**	

User Chromatograms

Integration Peak List

Peak	Start	RT	End	Height	Area	Area %	Signal To Noise
1	3.893	3.96	4.118	19.88	62.32	100	155

Fragmentor Voltage 120 **Collision Energy** 0 **Ionization Mode** ESI

Integration Peak List

Peak	Start	RT	End	Height	Area	Area %	Signal To Noise
1	3.779	3.956	4.374	217961	1888944	100	736.8

User Spectra

Fragmentor Voltage	Collision Energy	Ionization Mode
120	0	Esi

MS Formula Results: + Scan (3.956 min) Sub (2015112606.d)

m/z	Ion	Formula	Abundance
641.1699	(M+Na)+	C26 H34 Na O17	292094.8

Best	Formula (M)	Ion Formula	Score	Cross Sco	Mass	Calc Mass	Calc m/z	Diff (ppm)	Abs Diff (ppm)	Mass Match	Abund Match	Spacing Match	DBE
☑	C26 H34 O17	C26 H34 Na O17	99.52		618.1806	618.1796	641.1688	-1.69	1.69	99.91	99.35	98.94	10
☐	C23 H38 O17 S	C23 H38 Na O17 S	99.44		618.1807	618.183	641.1722	3.75	3.75	99.54	99	99.79	5
☐	C24 H34 N4 O13 S	C24 H34 N4 Na O13 S	99.25		618.1807	618.1843	641.1735	5.9	5.9	98.86	99.42	99.85	10
☐	C27 H30 N4 O13	C27 H30 N4 Na O13	99.21		618.1807	618.1809	641.1702	0.46	0.46	99.99	98.06	99.03	15
☐	C18 H38 N2 O19 S	C18 H38 N2 Na O19 S	98.77		618.1807	618.1789	641.1682	-2.77	2.77	99.75	96.21	99.87	1
☐	C27 H38 O12 S2	C27 H38 Na O12 S2	98.65		618.1807	618.1805	641.1697	-0.3	0.3	100	95.34	99.91	9
☐	C30 H34 O12 S	C30 H34 Na O12 S	98.6		618.1807	618.1771	641.1663	-5.75	5.75	98.92	97.16	99.71	14
☐	C28 H34 N4 O8 S2	C28 H34 N4 Na O8 S2	98.5		618.1807	618.1818	641.171	1.85	1.85	99.89	94.97	99.95	14
☐	C31 H30 N4 O8 S	C31 H30 N4 Na O8 S	98.42		618.1807	618.1784	641.1677	-3.6	3.6	99.57	95.38	99.76	19
☐	C36 H30 N2 O6 S	C36 H30 N2 Na O6 S	97.07		618.1807	618.1825	641.1717	2.92	2.92	99.72	90.51	99.65	23
☐	C39 H26 N2 O6	C39 H26 N2 Na O6	95.47		618.1806	618.1791	641.1683	-2.53	2.53	99.79	85.54	98.76	28
☐	C40 H30 N2 O S2	C40 H30 N2 Na O S2	95.32		618.1807	618.18	641.1692	-1.13	1.13	99.96	83.82	99.84	27
☐	C44 H26 O4	C44 H26 Na O4	93.51		618.1806	618.1831	641.1723	3.98	3.98	99.48	79.28	98.64	32

Figure S21. HRESIMS spectrum of compound **3**.

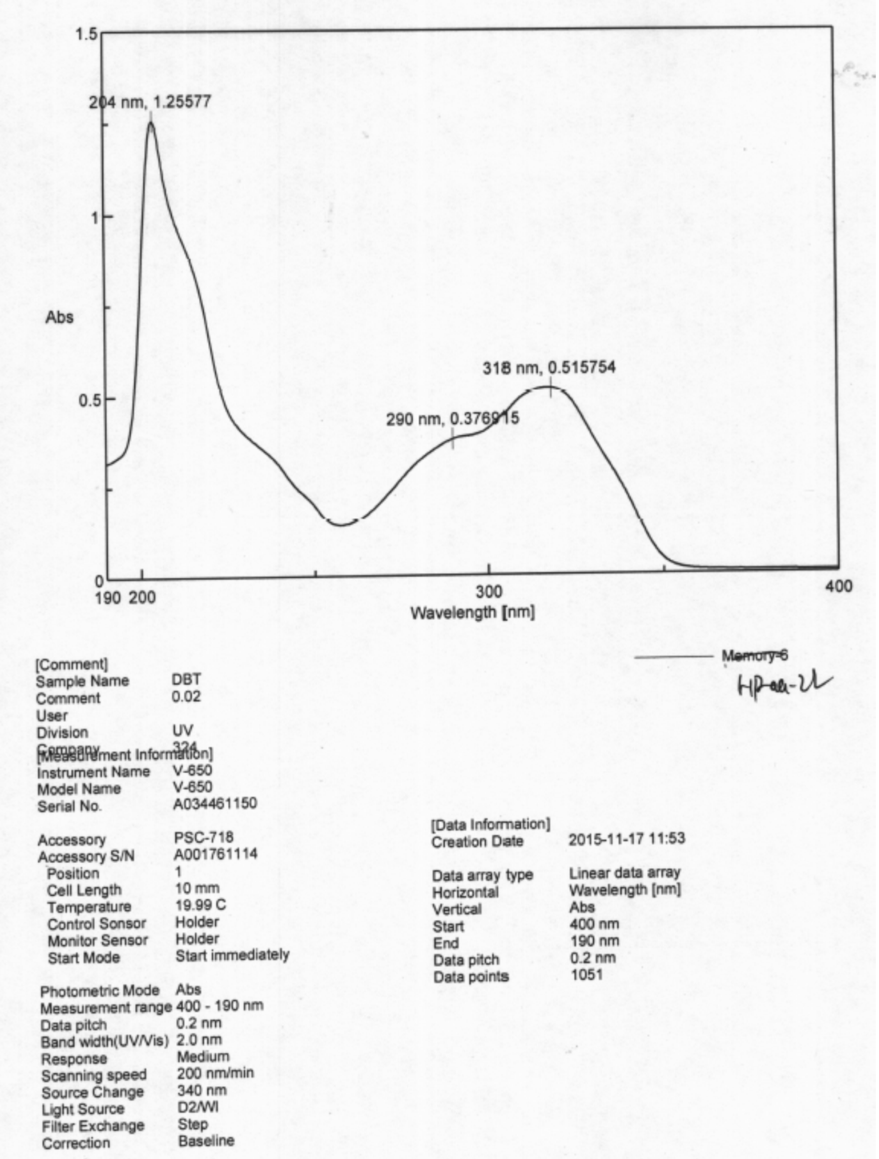

Figure S22. UV spectrum of compound **3**.

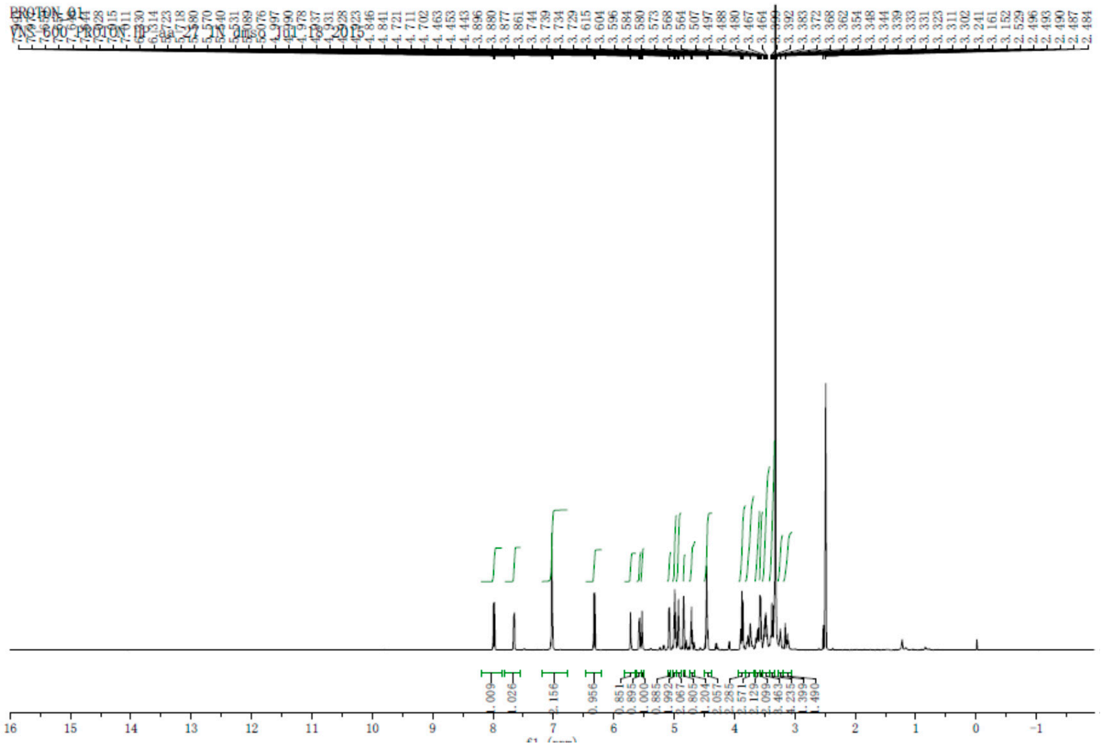

Figure S23. ¹H-NMR spectrum of compound **3**.

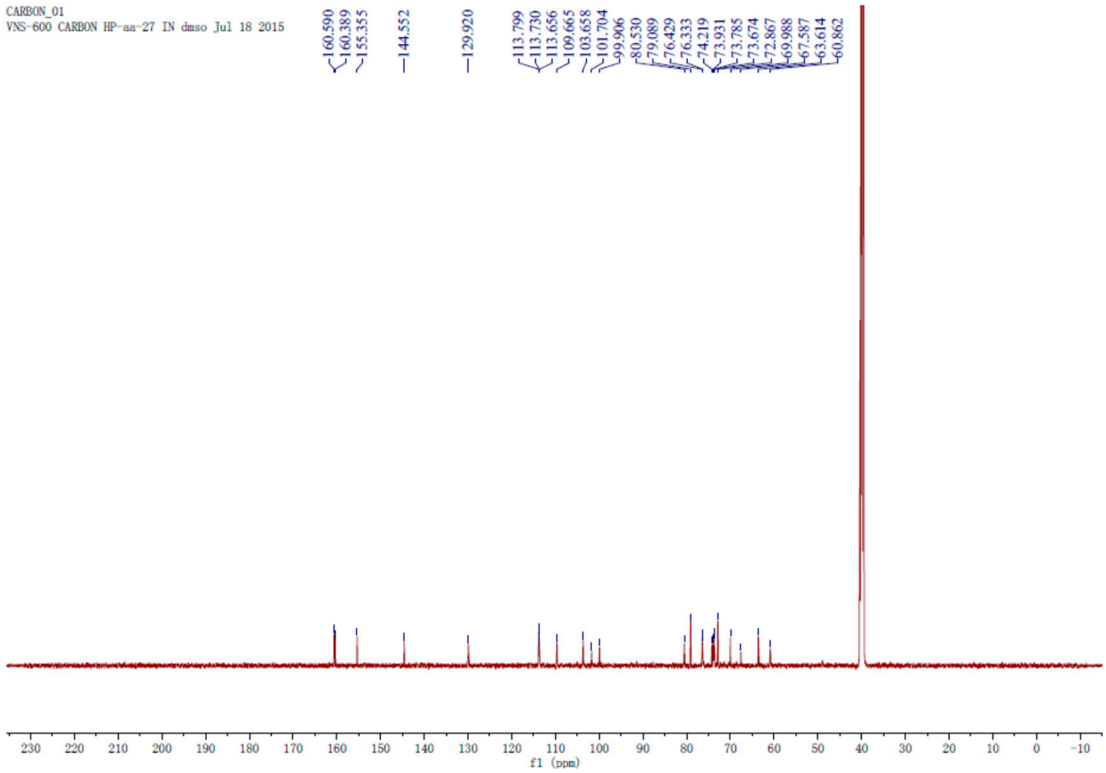

Figure S24. ¹³C-NMR spectrum of compound **3**.

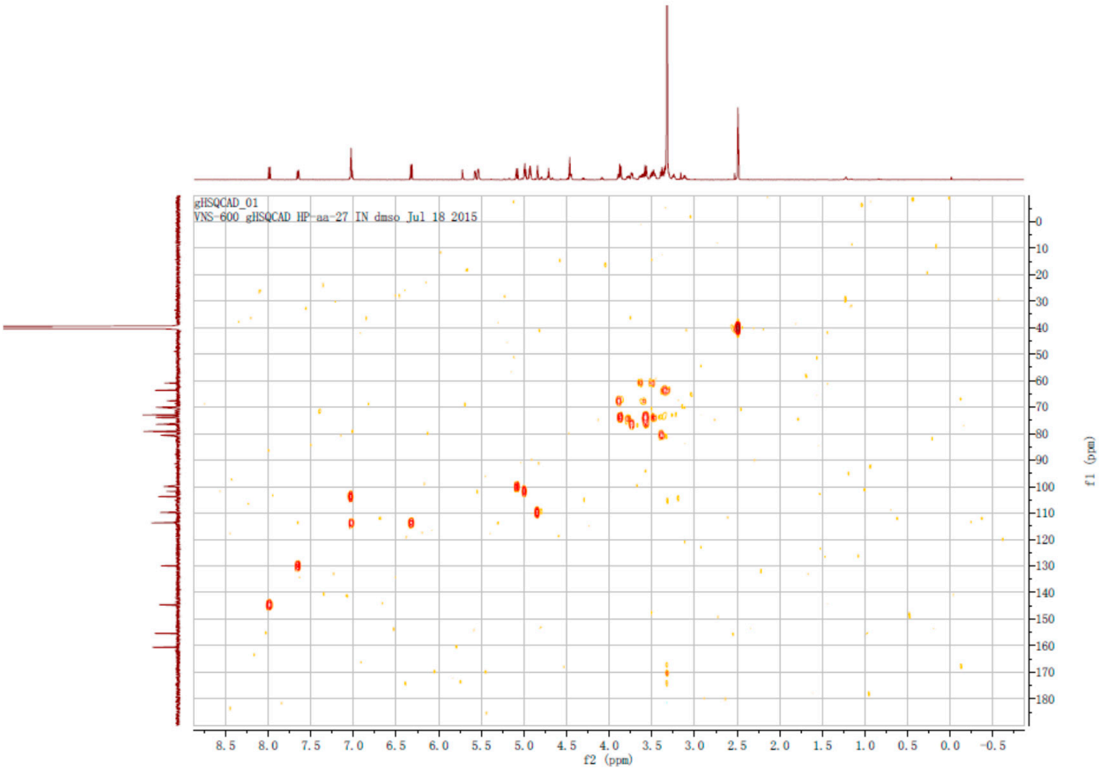

Figure S25. HSQC spectrum of compound **3**.

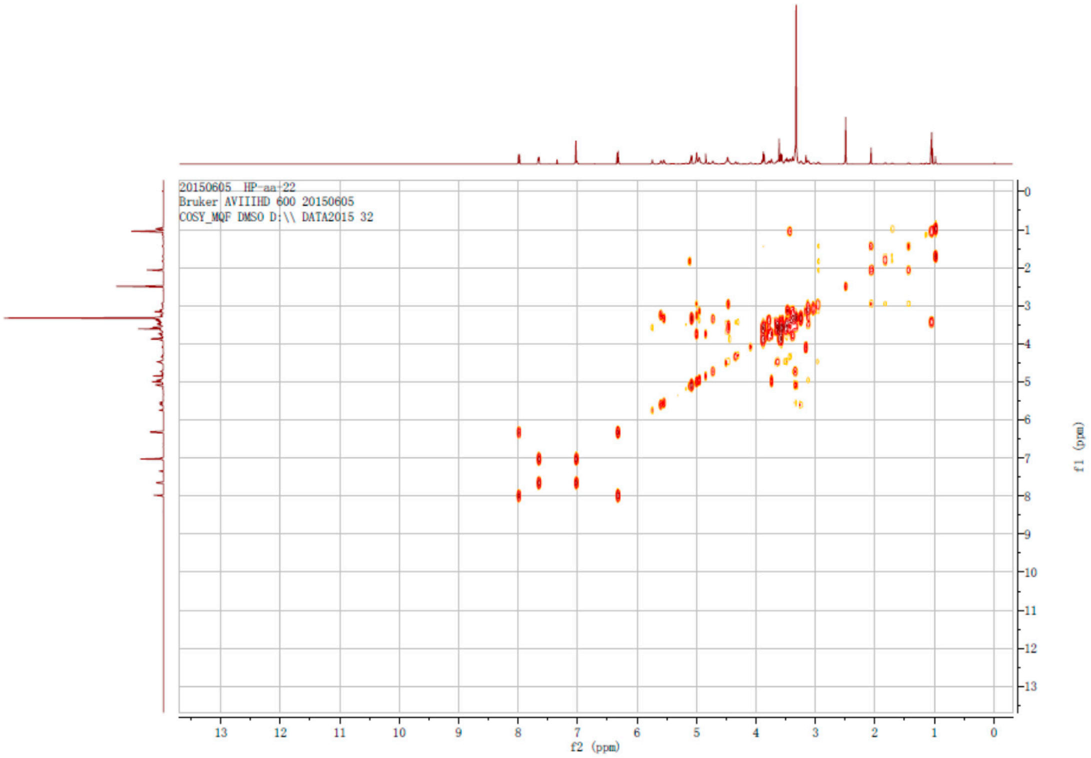

Figure S26. gCOSY spectrum of compound **3**.

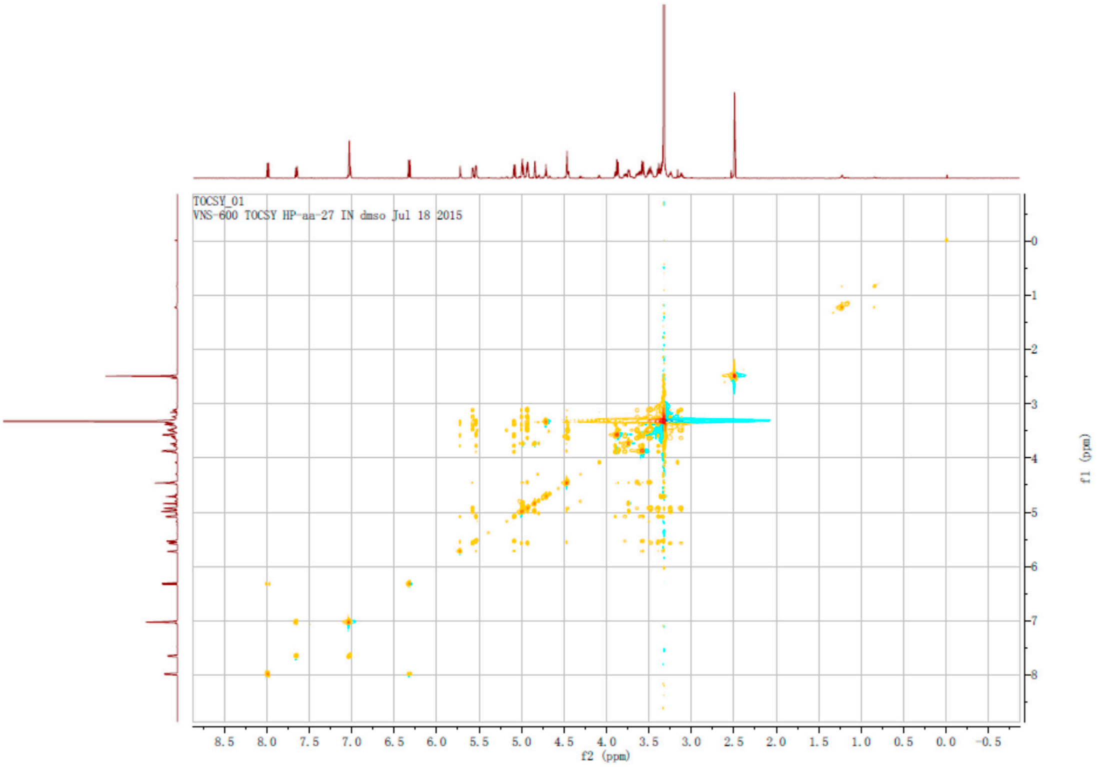

Figure S27. TOCSY spectrum of compound **3**.

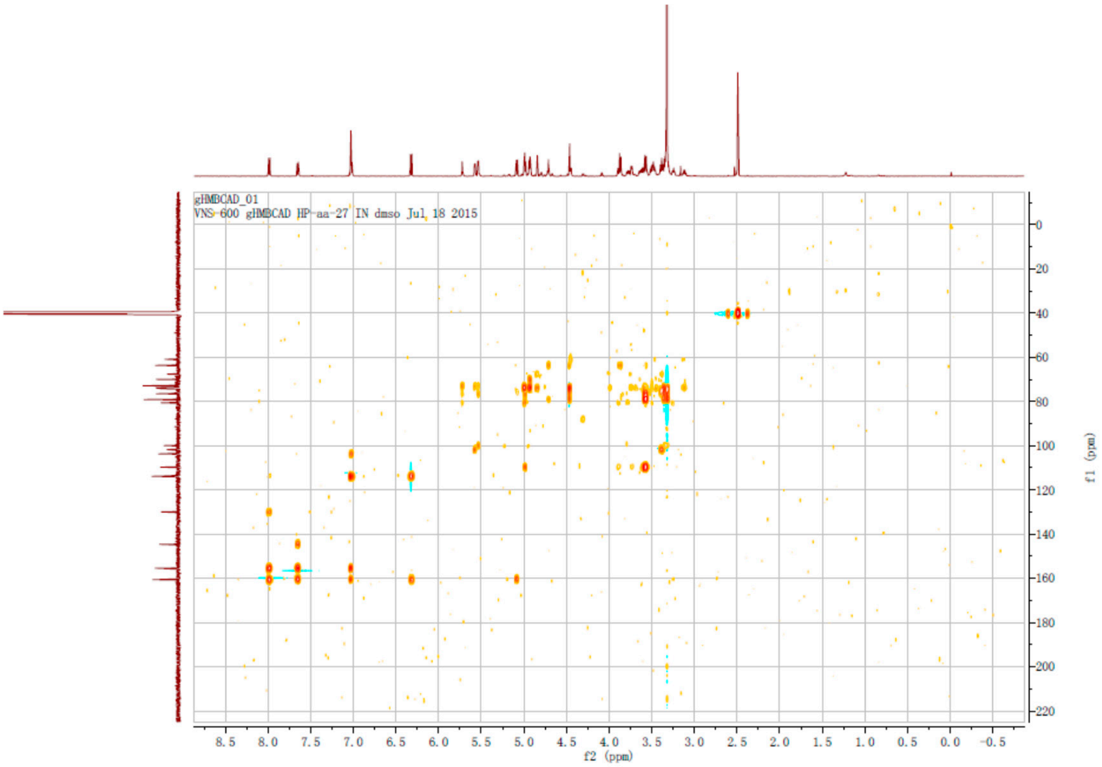

Figure S28. HMBC spectrum of compound **3**.

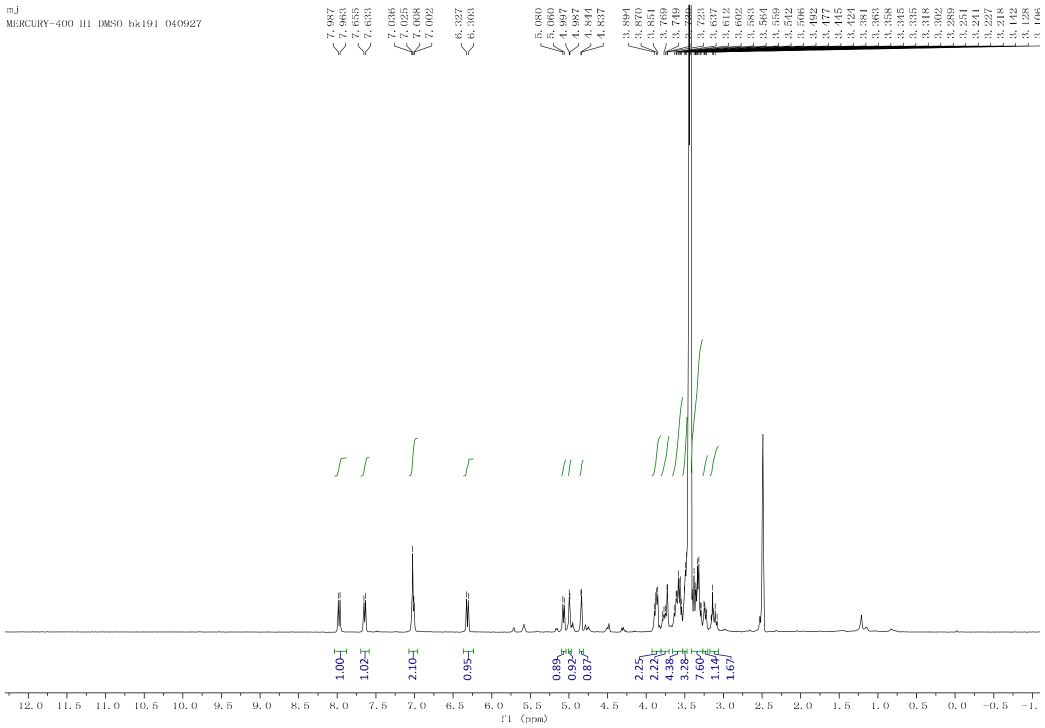

Figure S29. ^{1}H-NMR spectrum (in DMSO-d_6 + D_2O) of compound **3**.

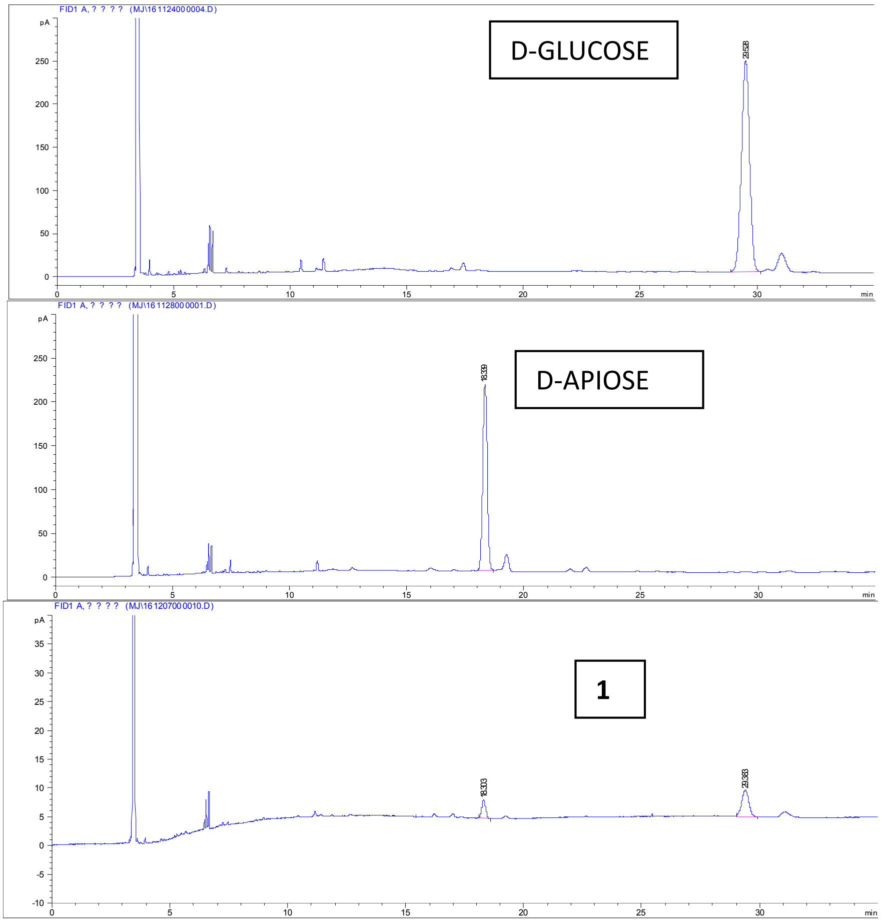

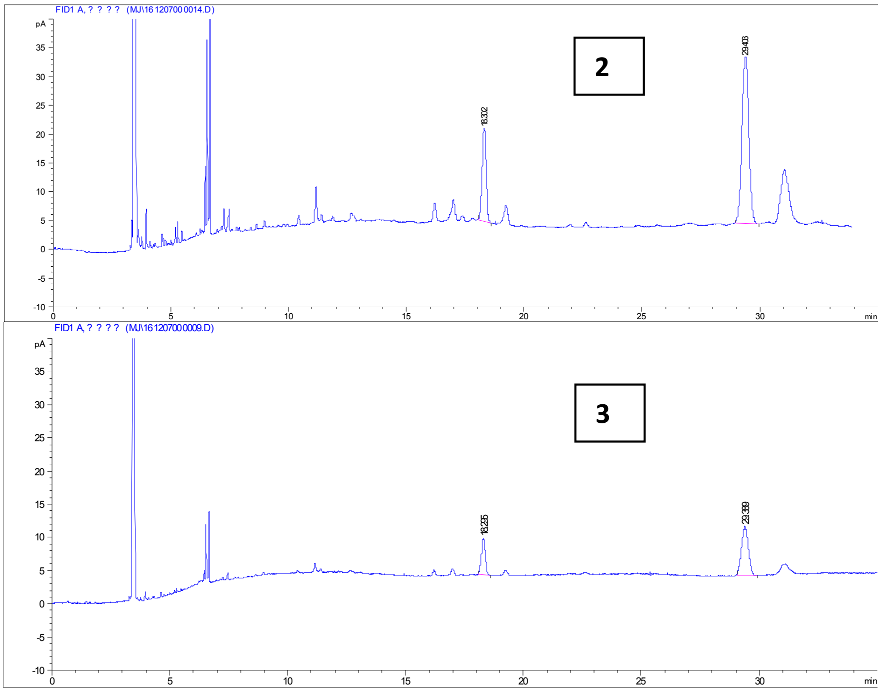

Figure S30. GC spectra of compounds **1–3**.
